# Supplementary material for: Plasma from patients with pulmonary embolism show aggregates that reduce after anticoagulation
Source: Commun Med (Lond). 2023 Jan 28;3:12. doi: 10.1038/s43856-023-00242-8 (PMC9883810; doi:10.1038/s43856-023-00242-8)
Supplement: Supplementary file 1 — Supplementary Data 1 [file 43856_2023_242_MOESM1_ESM.pdf]

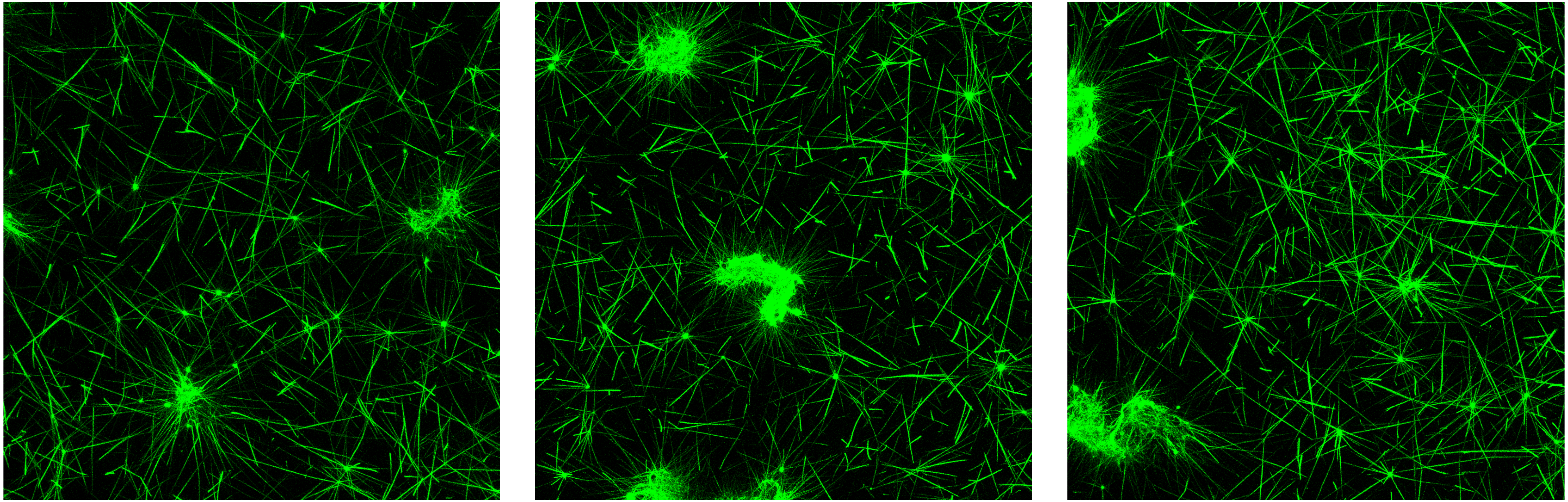

11I (Pre-treatment)

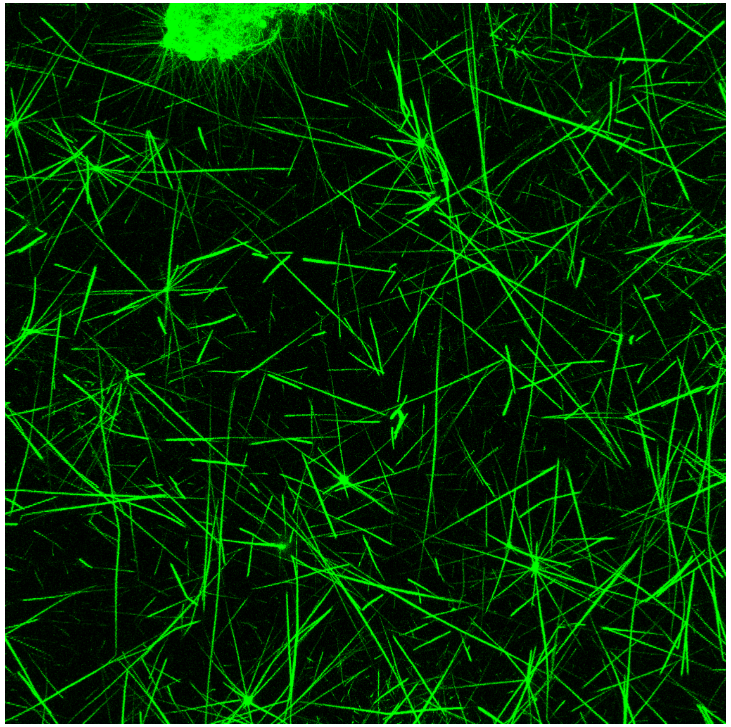

12I (Pre-treatment)

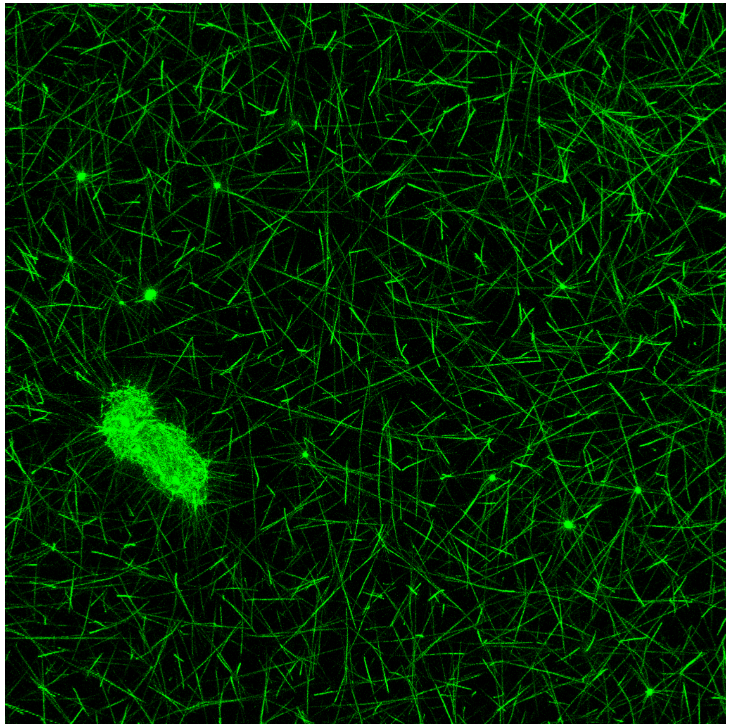

13I (Pre-treatment)

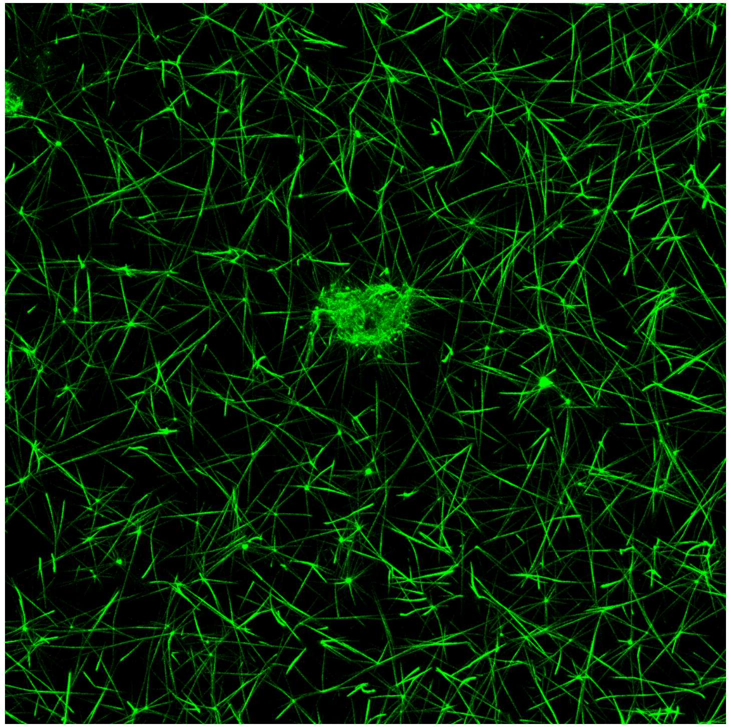
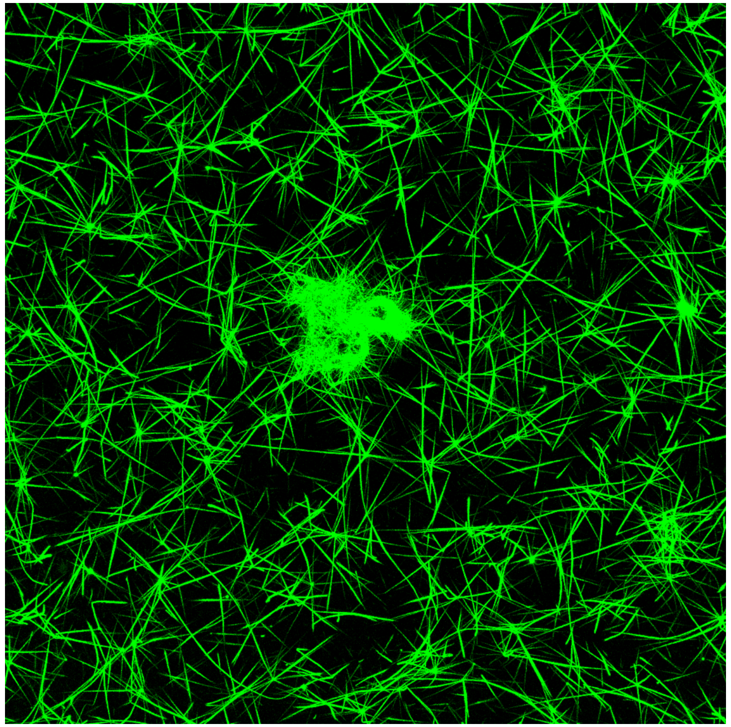
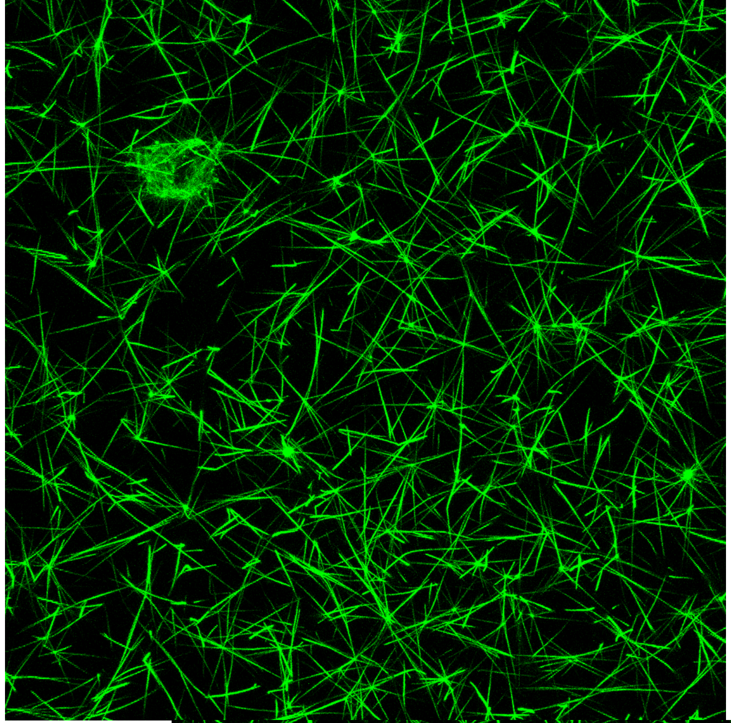
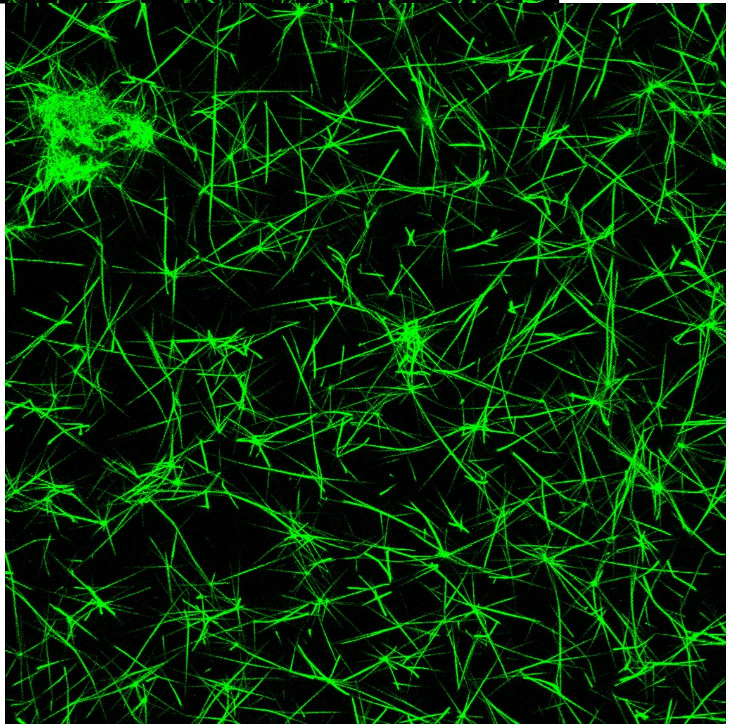

17I (Pre-treatment)

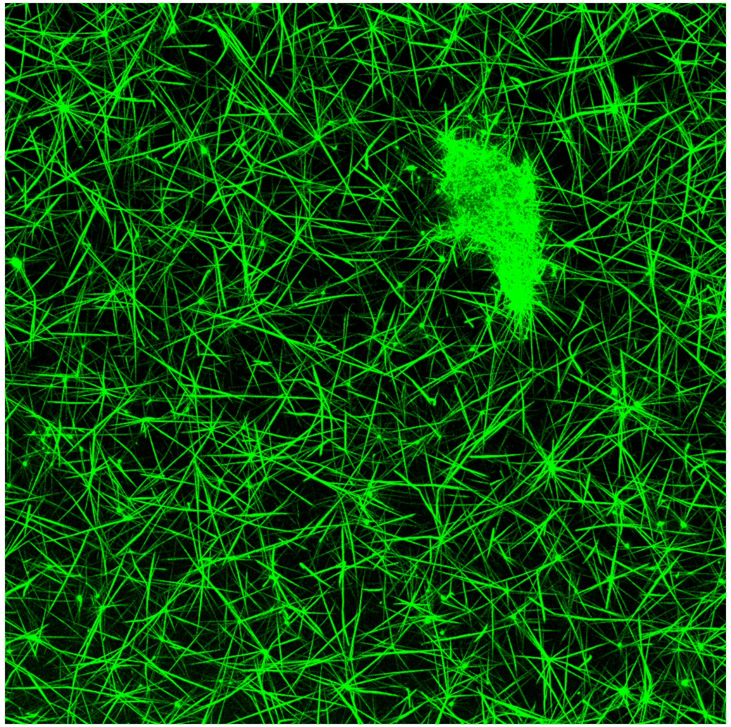
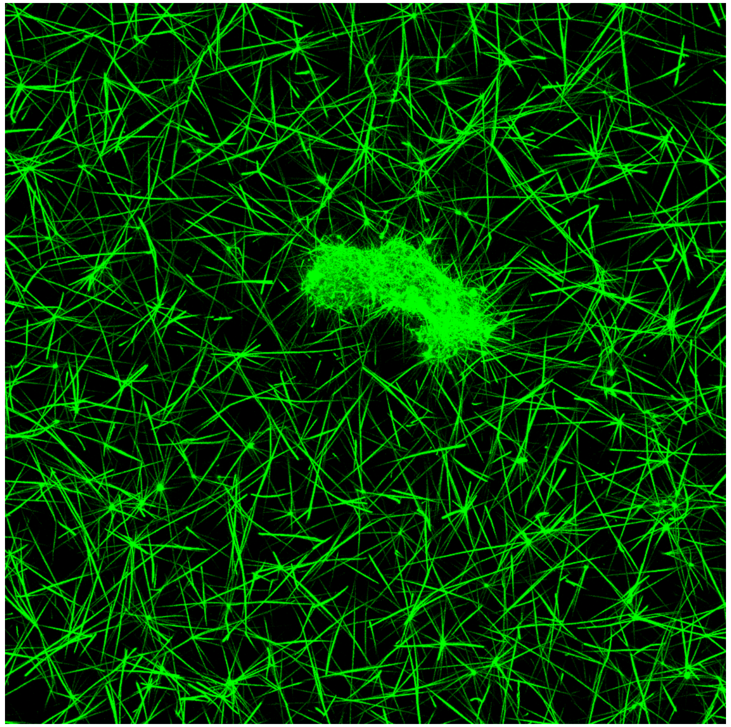
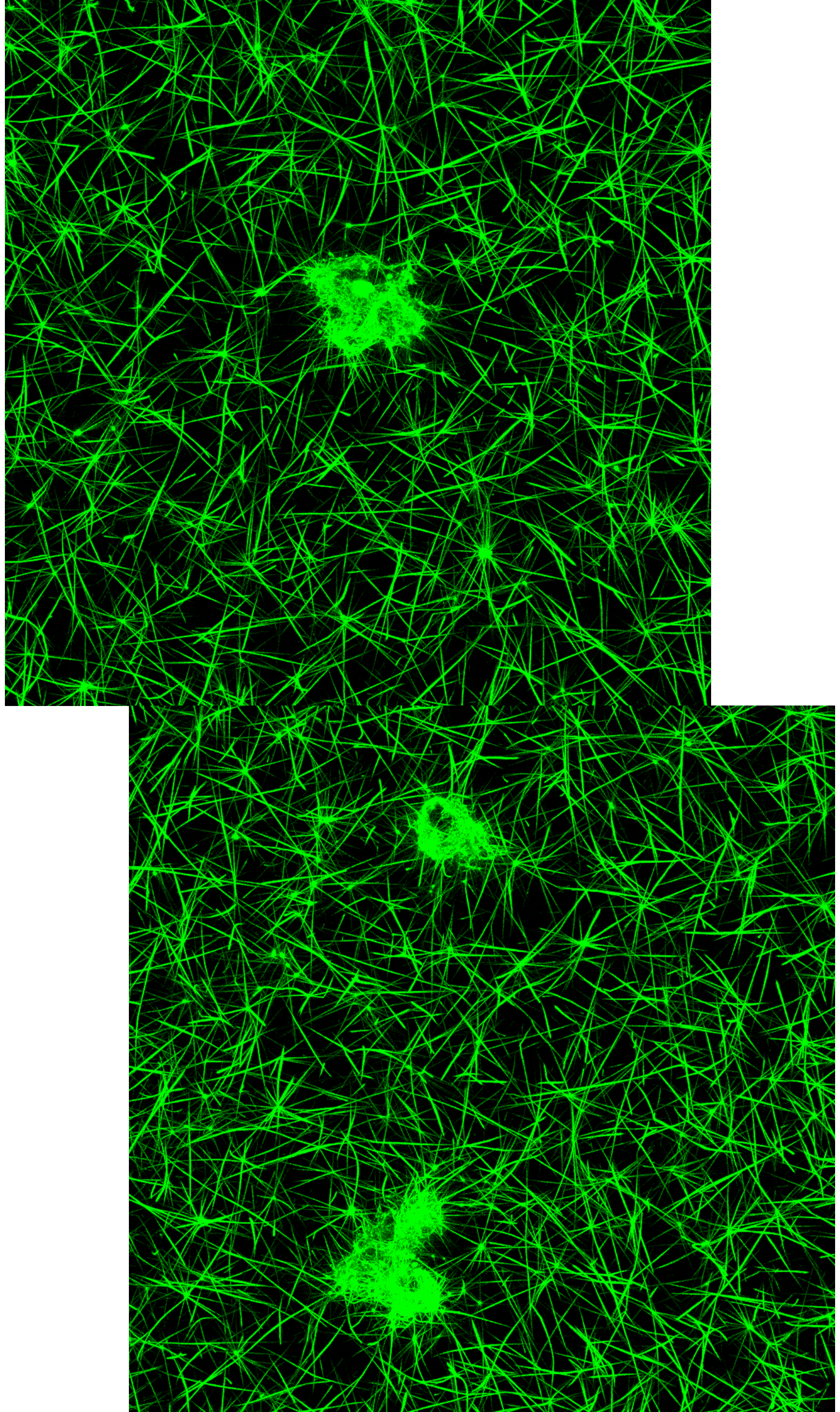

22I (Pre-treatment)

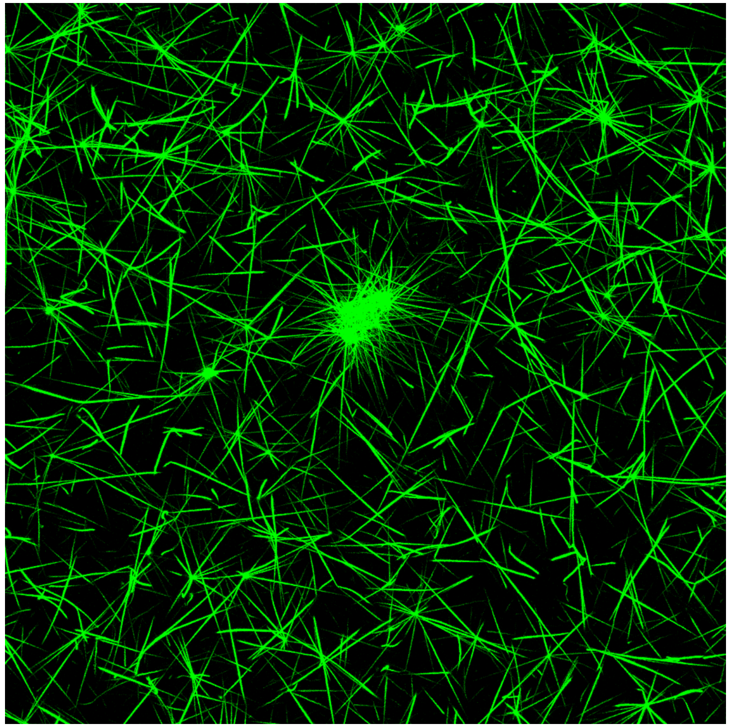
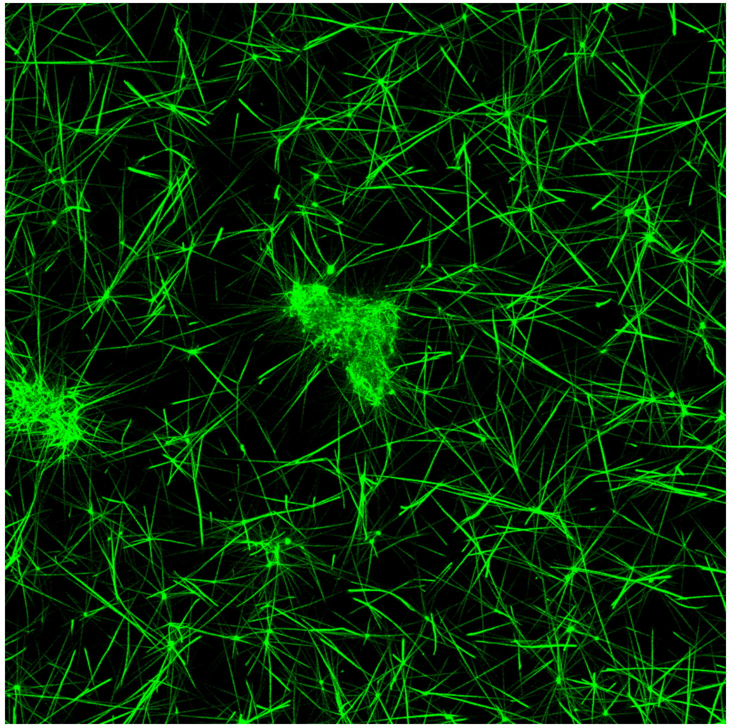
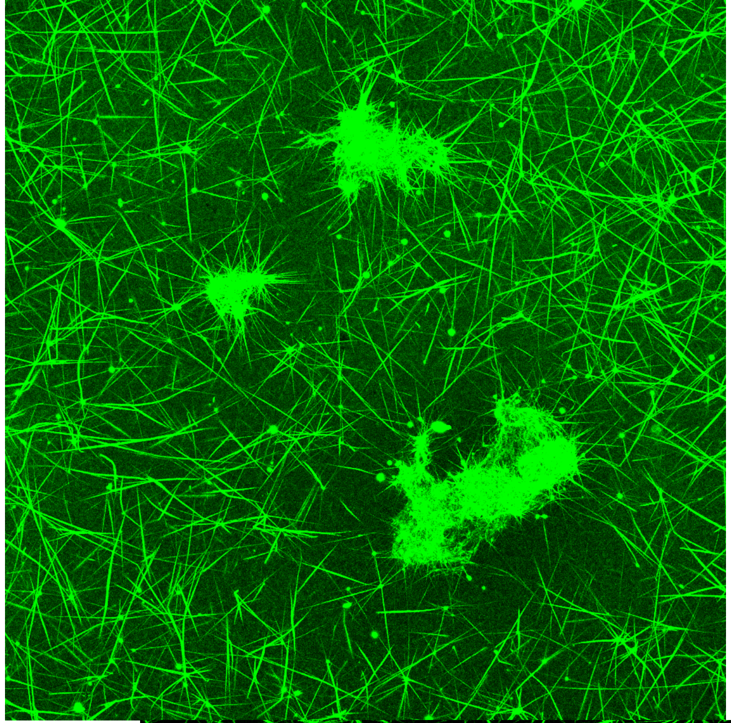
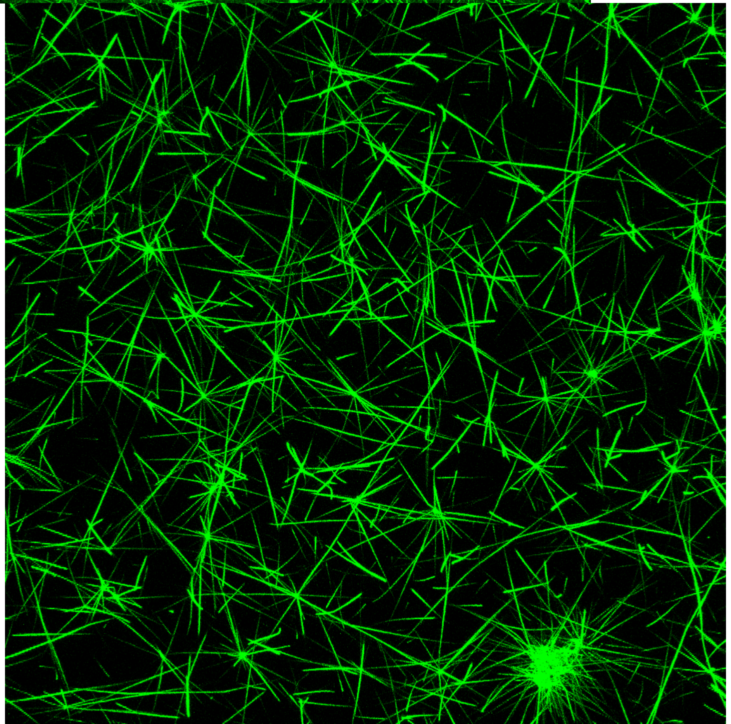

23I (Pre-treatment)

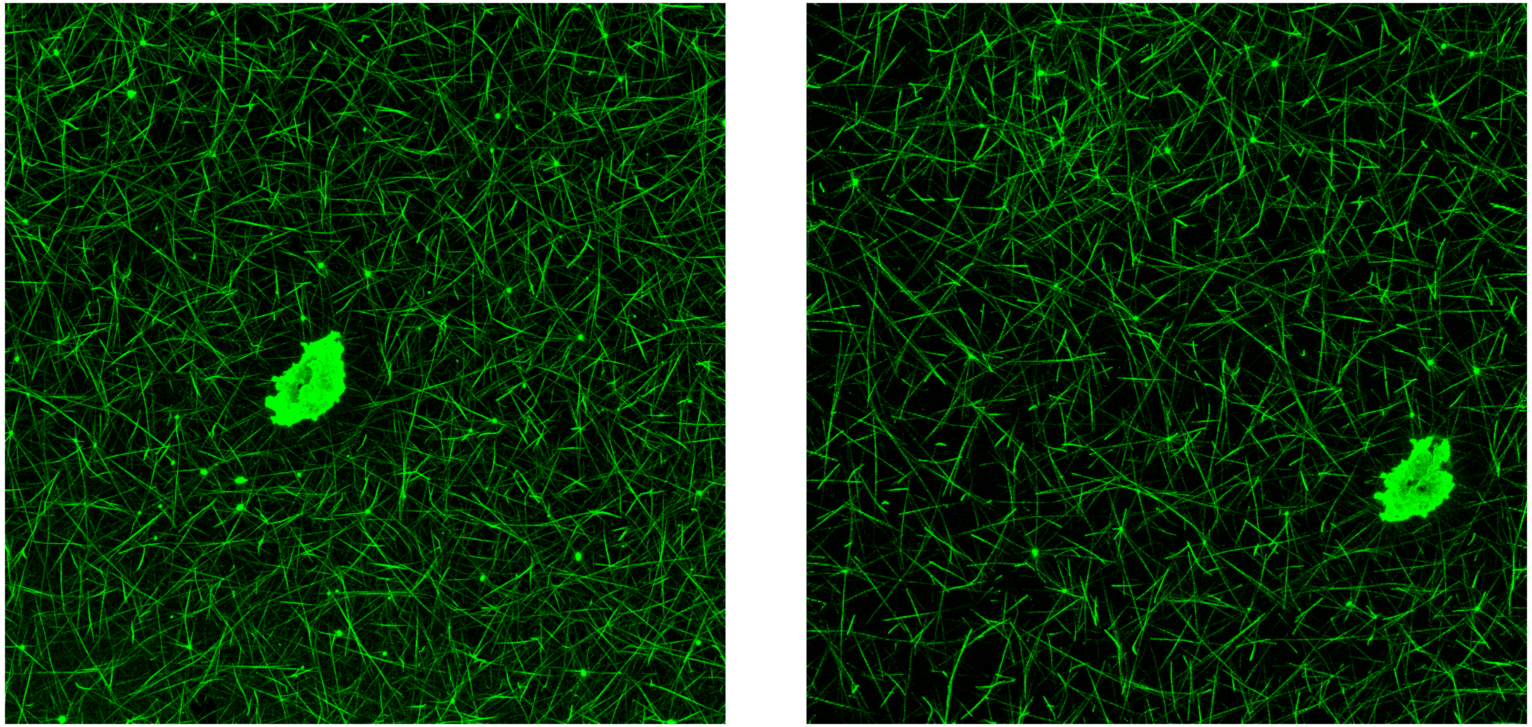

32I (Pre-treatment)

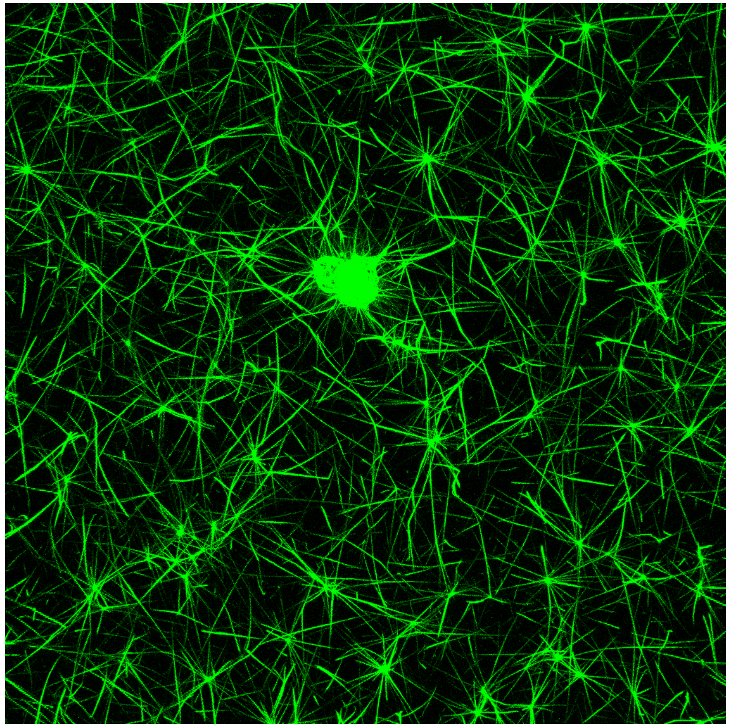

22II (Post-treatment)

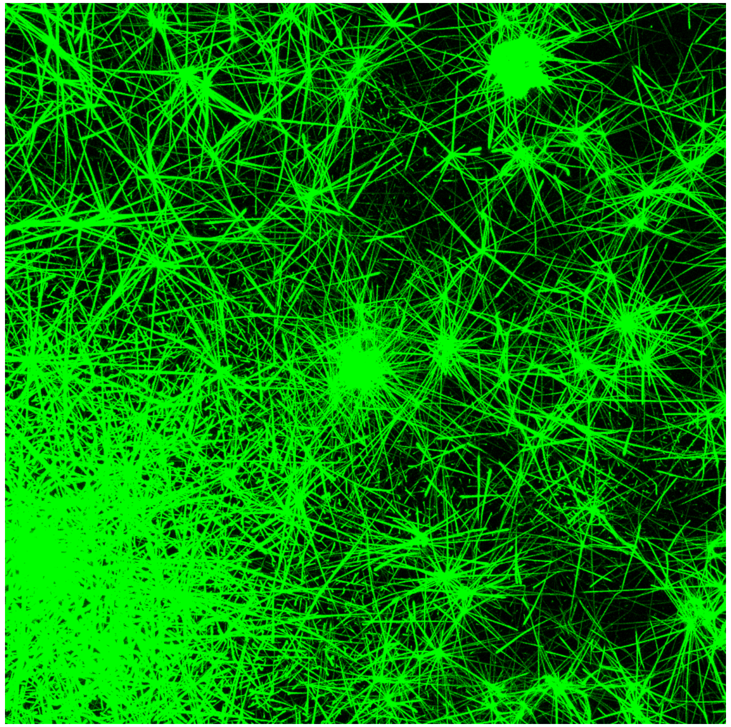

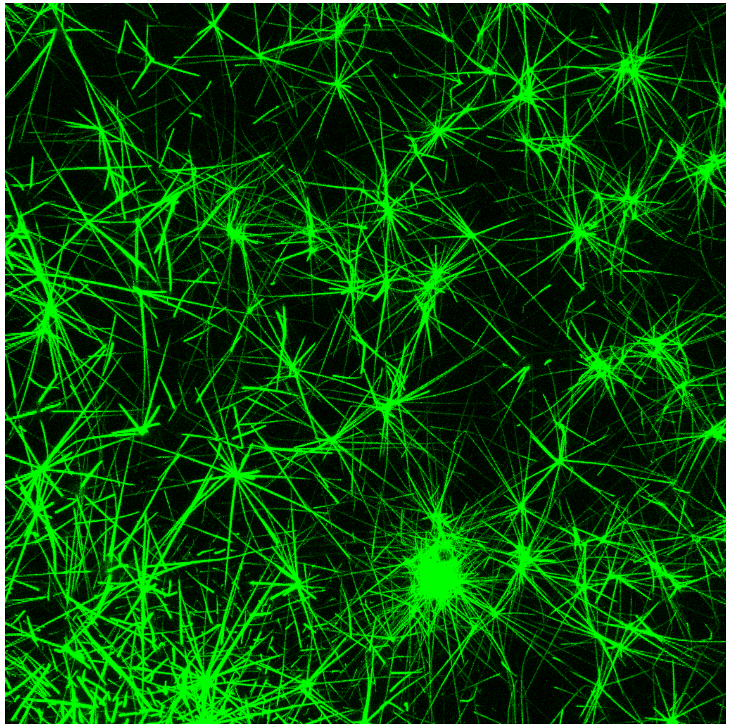

39I (Pre-treatment)

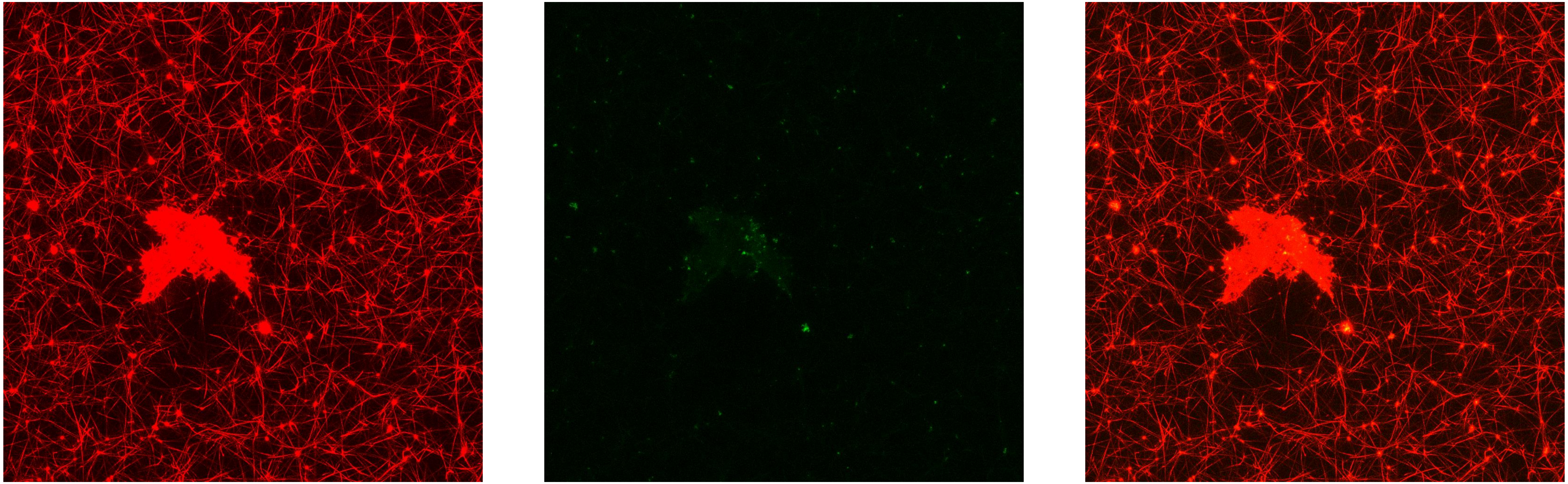

FGN PLT Merge

11I (Pre-treatment)

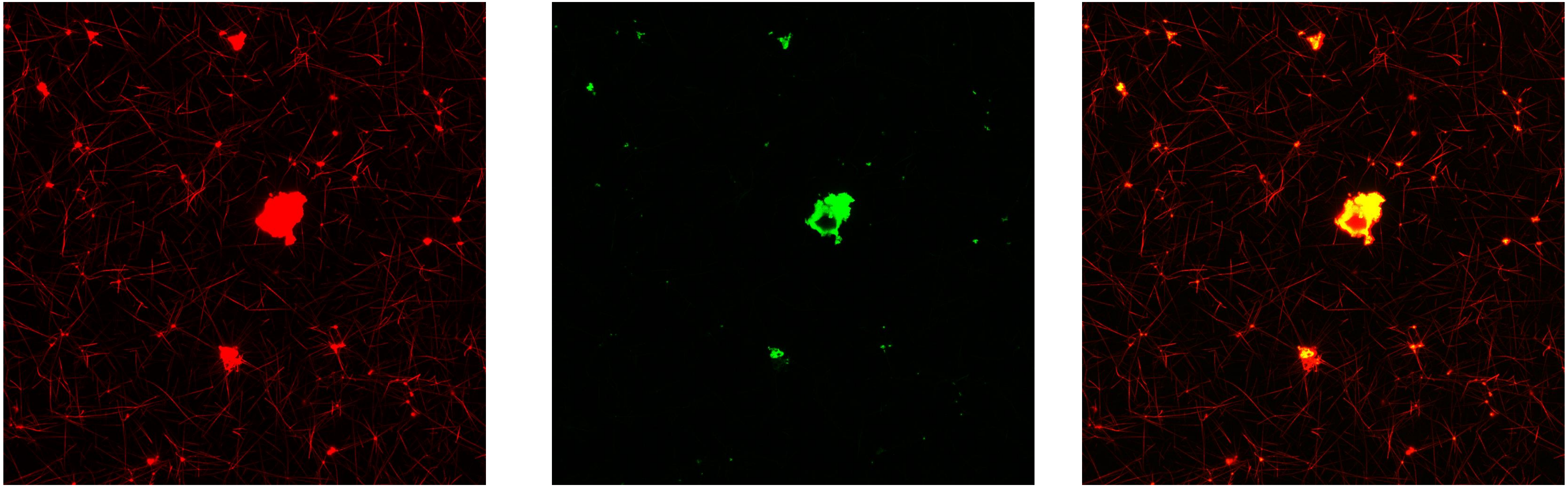

FGN PLT Merge

11I (Pre-treatment)

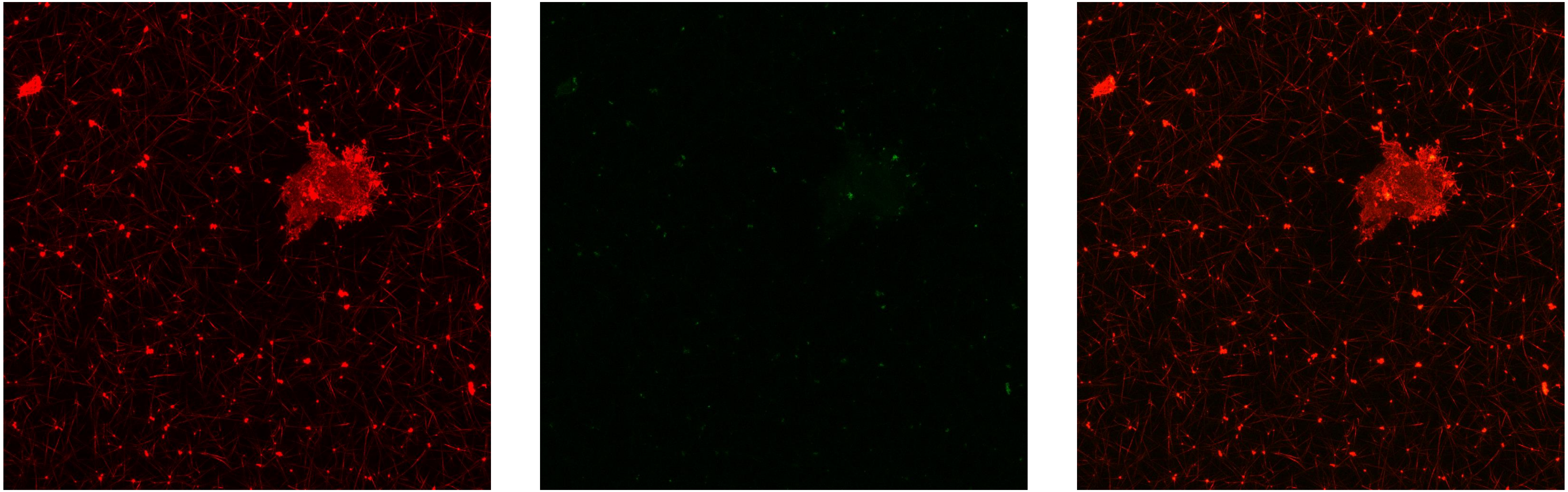

FGN PLT Merge

22I (Pre-treatment)

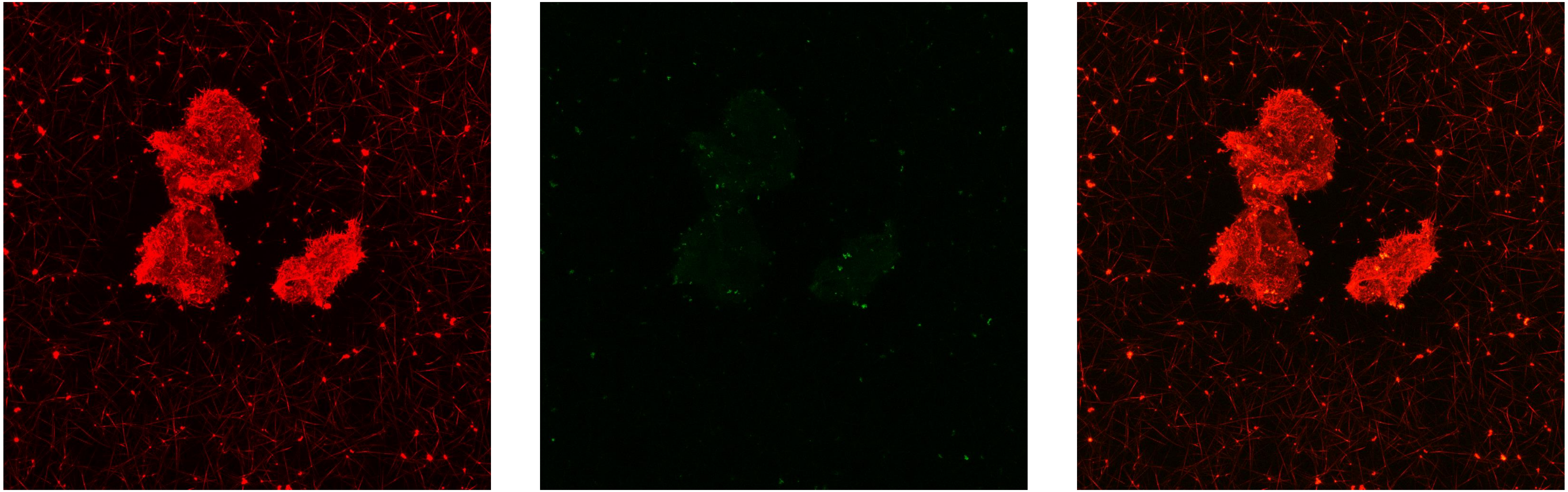

FGN PLT Merge

22I (Pre-treatment)

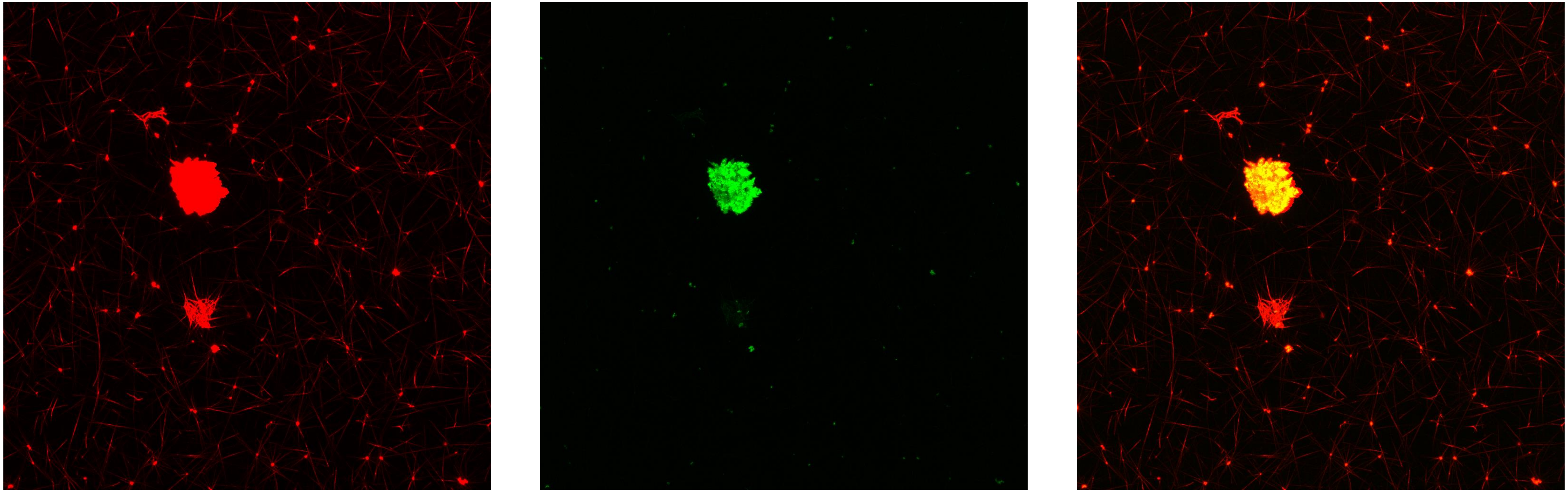

FGN PLT Merge

22II (Post-treatment)

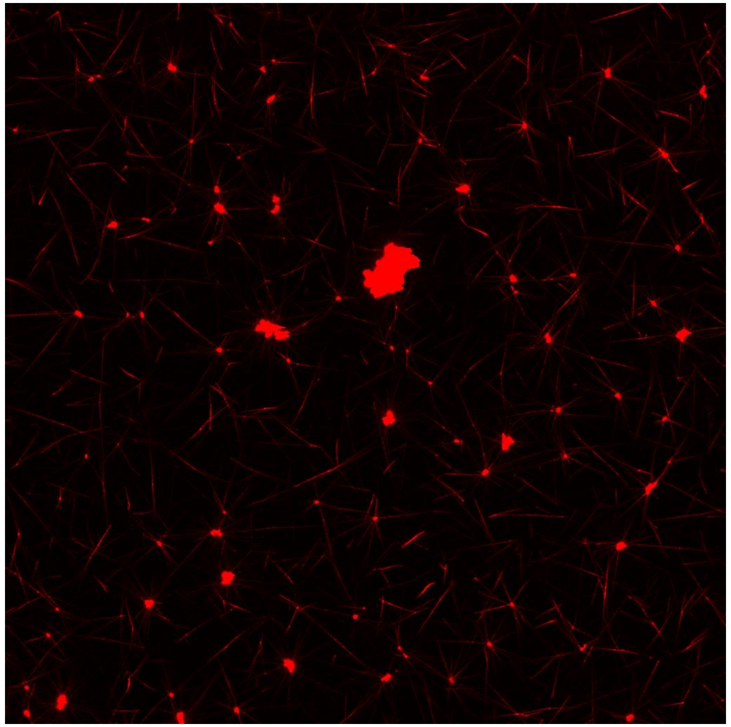

FGN

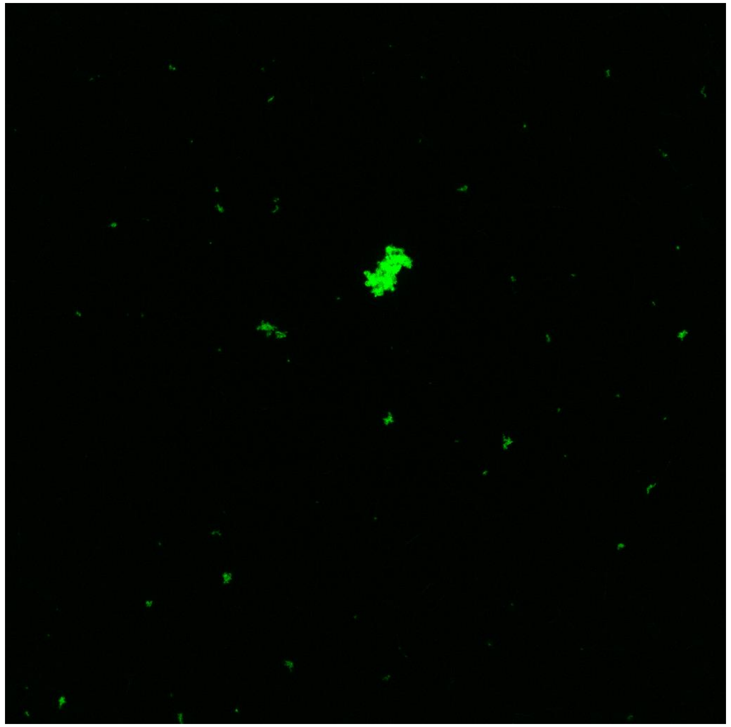

PLT

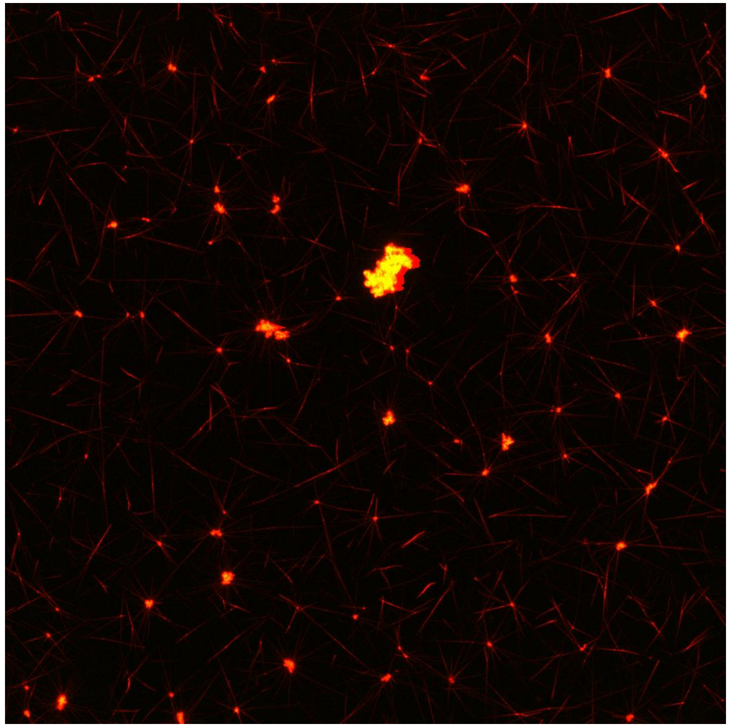

Merge

22II (Post-treatment)

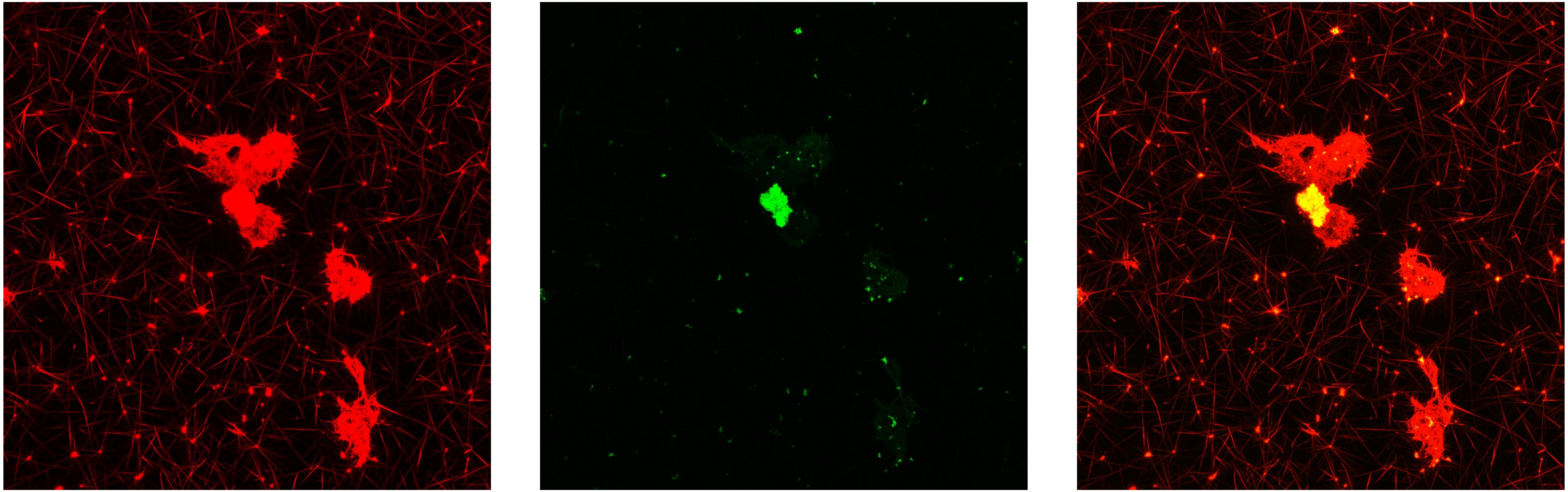

FGN PLT Merge

23I (Pre-treatment)

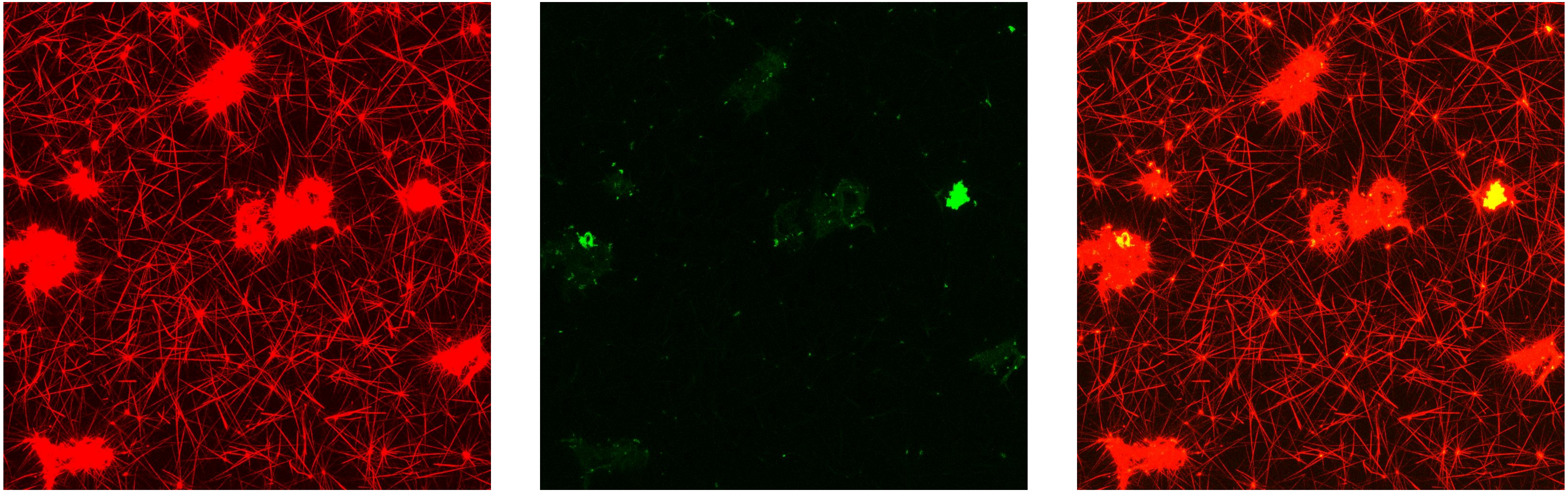

FGN PLT Merge

23I (Pre-treatment)

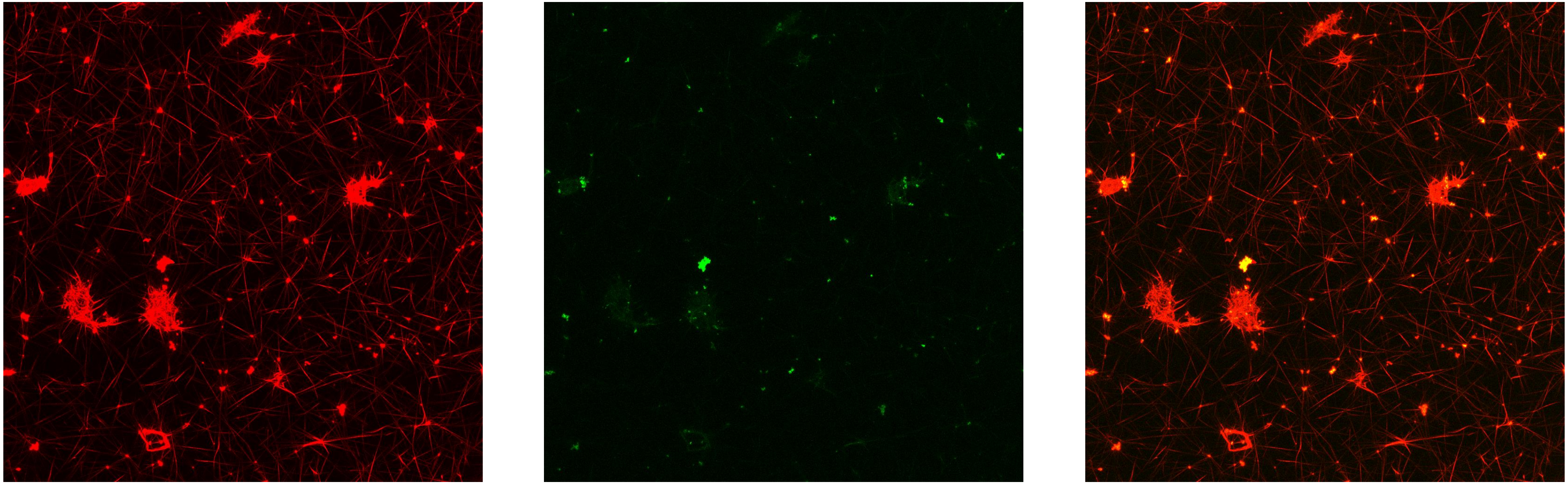

FGN PLT Merge

23I (Pre-treatment)

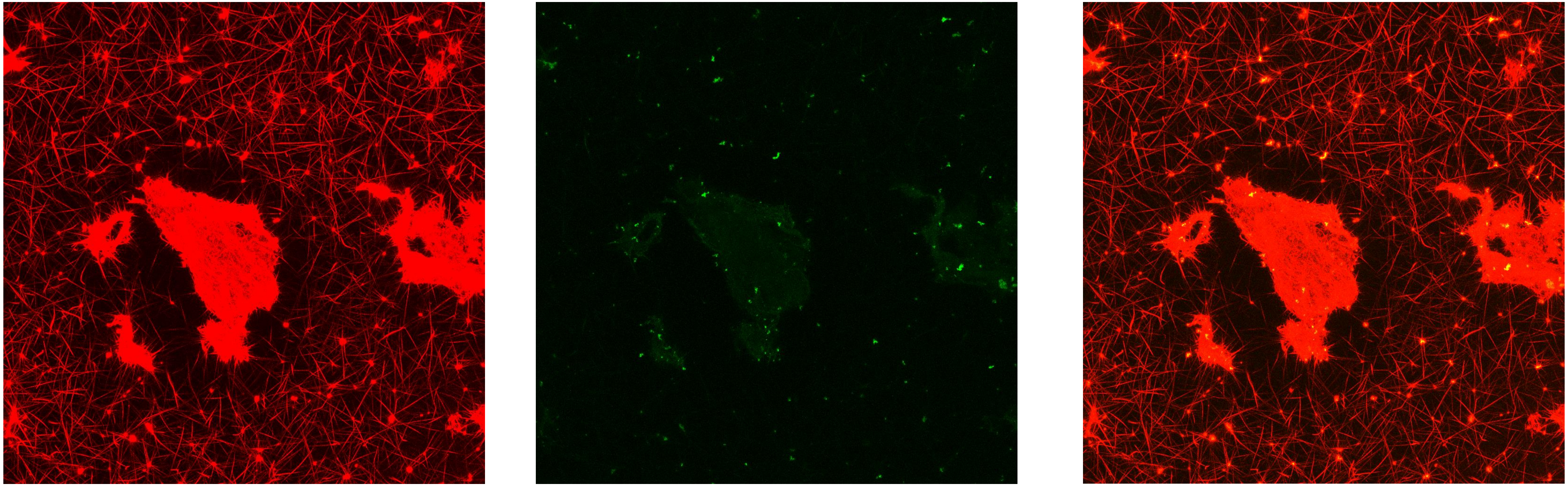

FGN PLT Merge

23I (Pre-treatment)

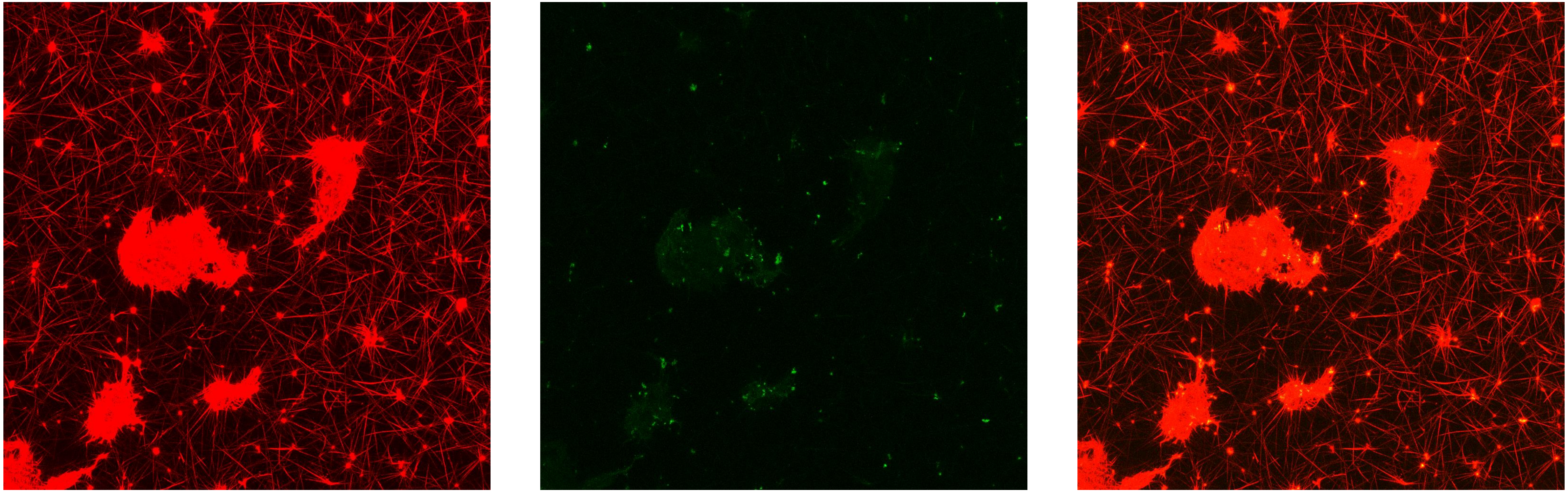

FGN PLT Merge

23I (Pre-treatment)

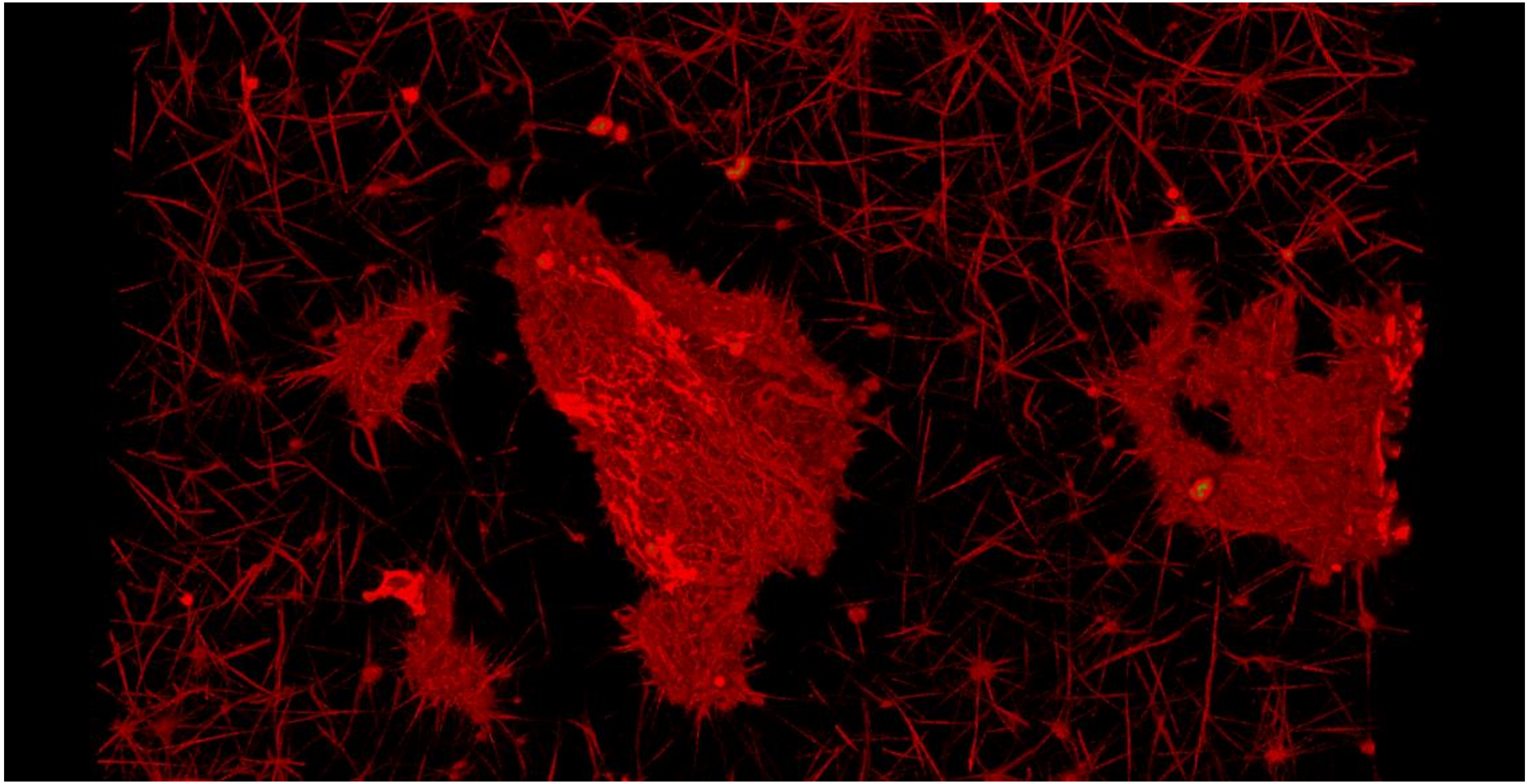

Merge (3D Render)

23I (Pre-treatment)

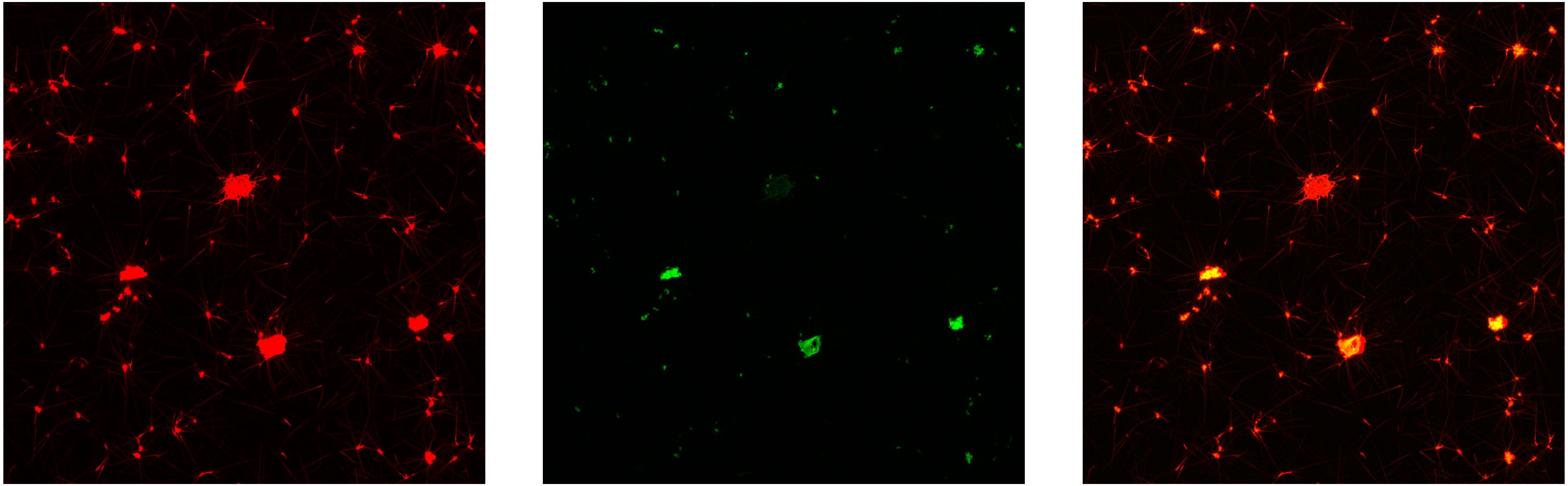

FGN PLT Merge

39I (Pre-treatment)

The following are No Thrombin Added

NO IIa

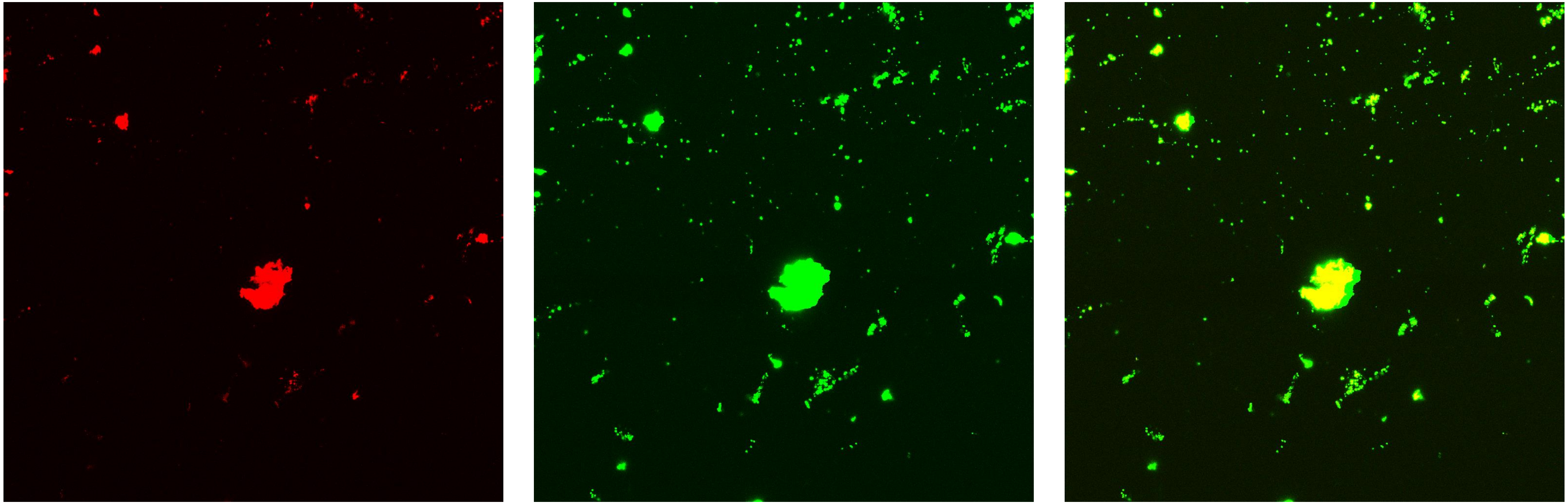

FGN PLT Merge

11I (Pre-treatment)

NO IIa

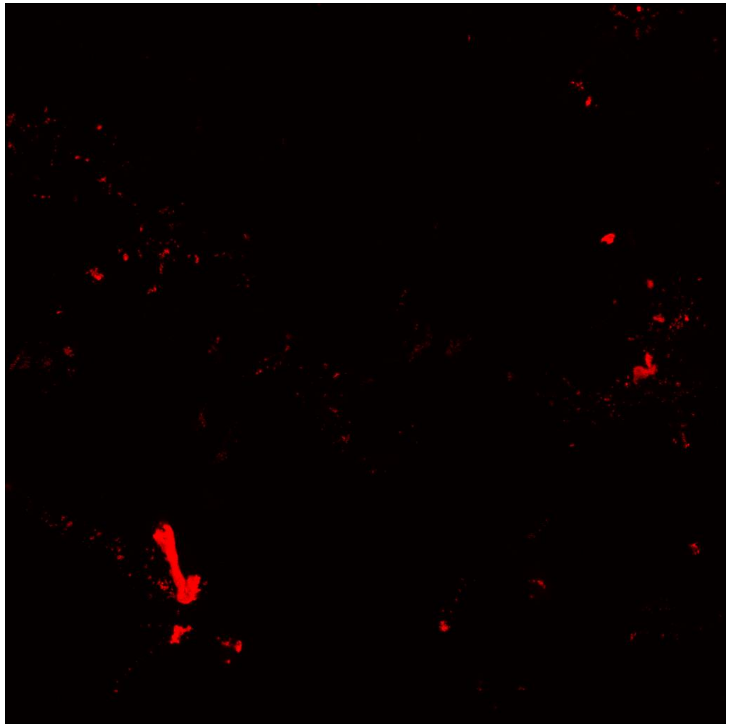

FGN

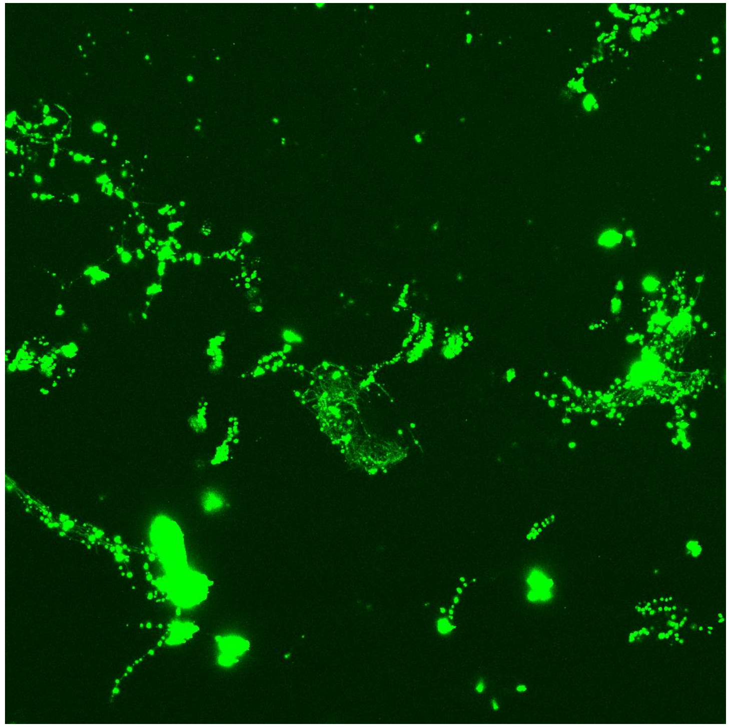

PLT

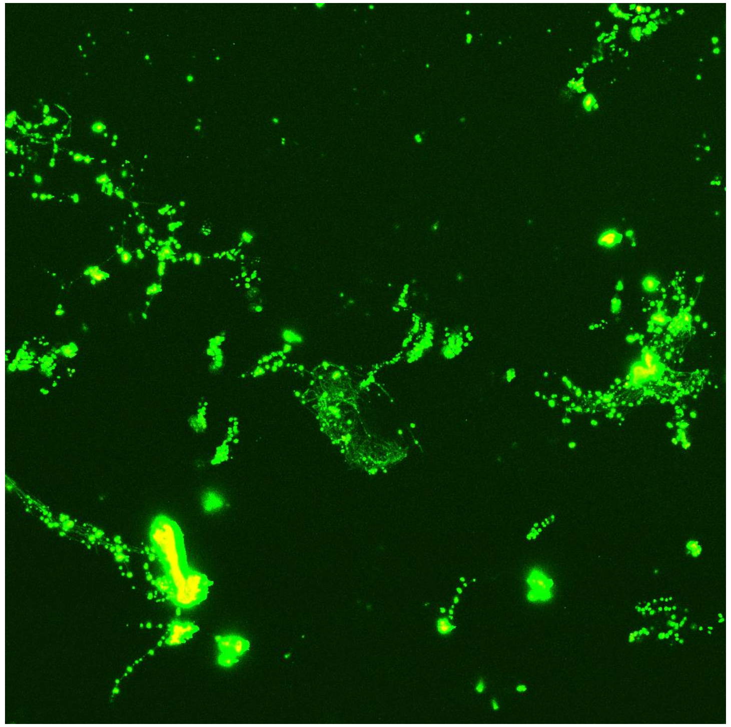

Merge

11I (Pre-treatment)

NO IIa

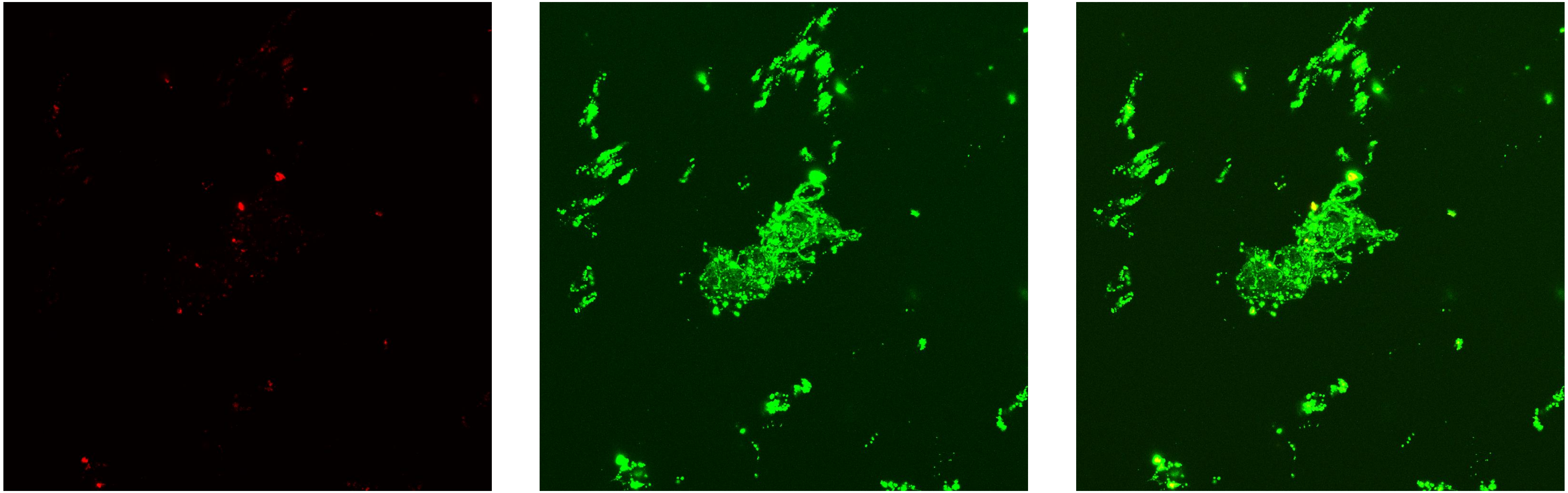

FGN PLT Merge

11I (Pre-treatment)

NO IIa

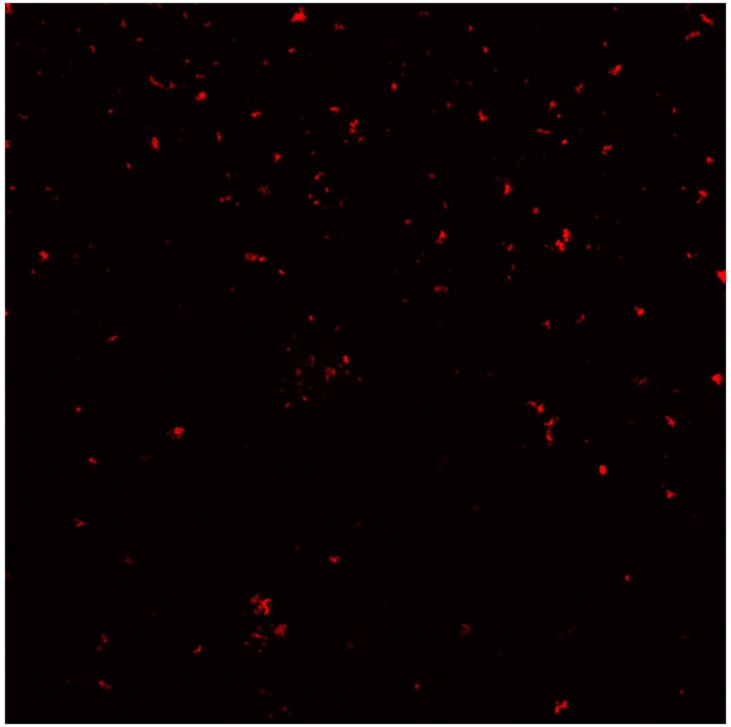

FGN

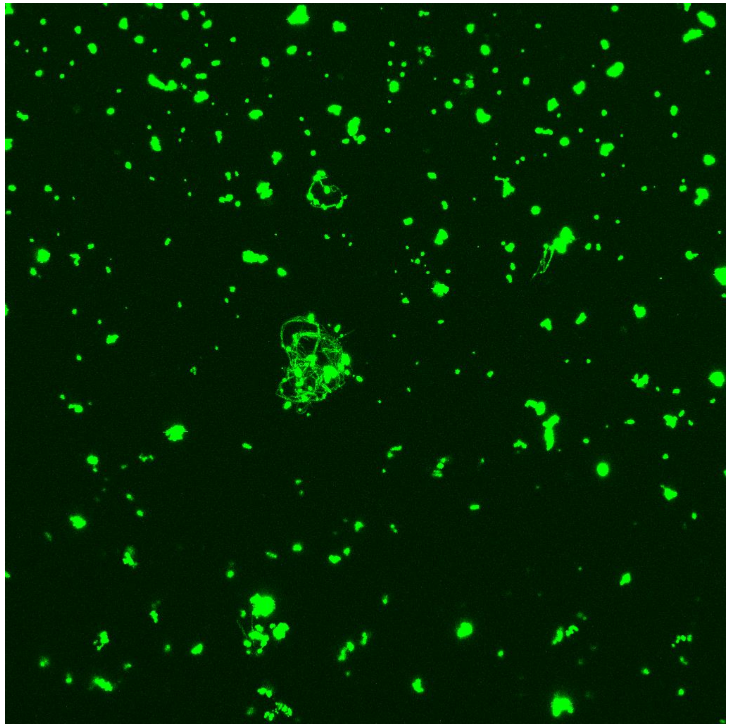

PLT

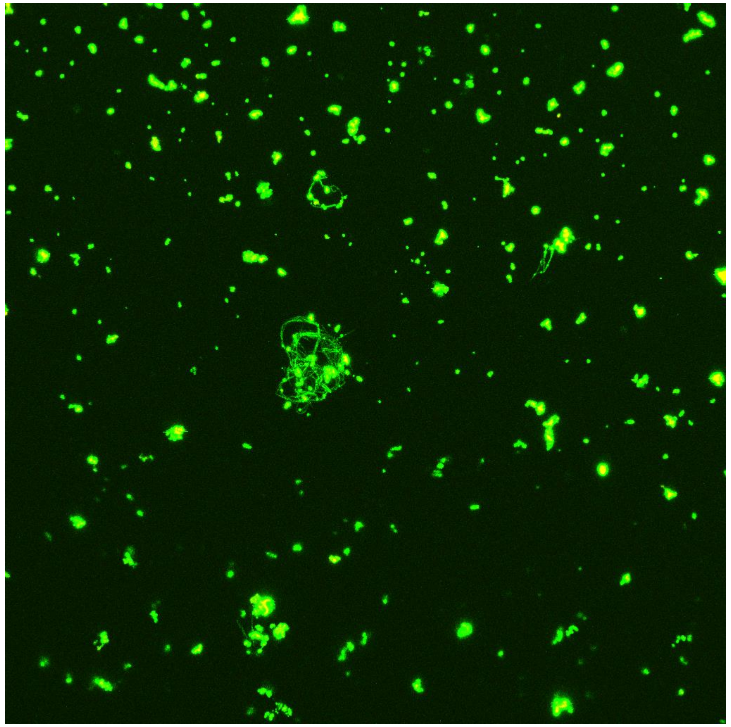

Merge

22I (Pre-treatment)

NO IIa

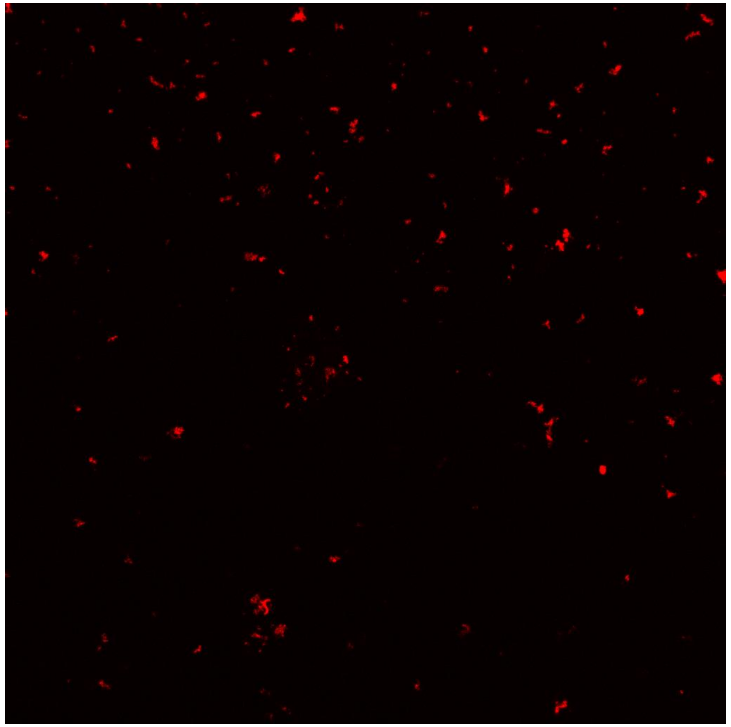

FGN

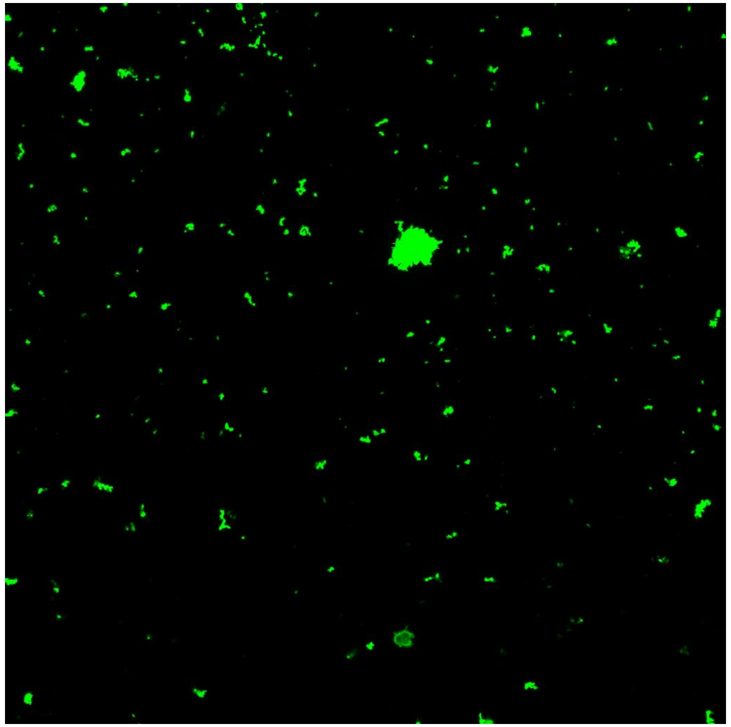

PLT

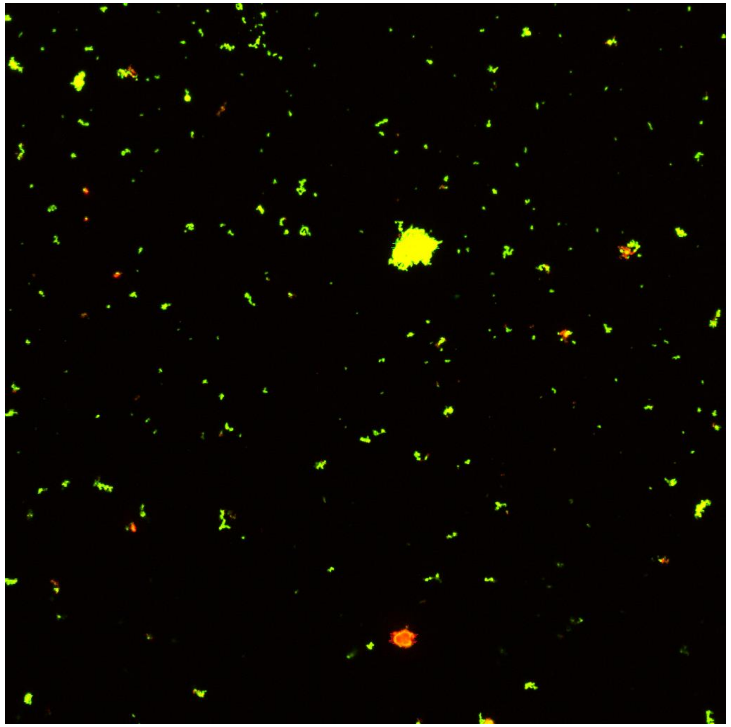

Merge

22I (Pre-treatment)

NO IIa

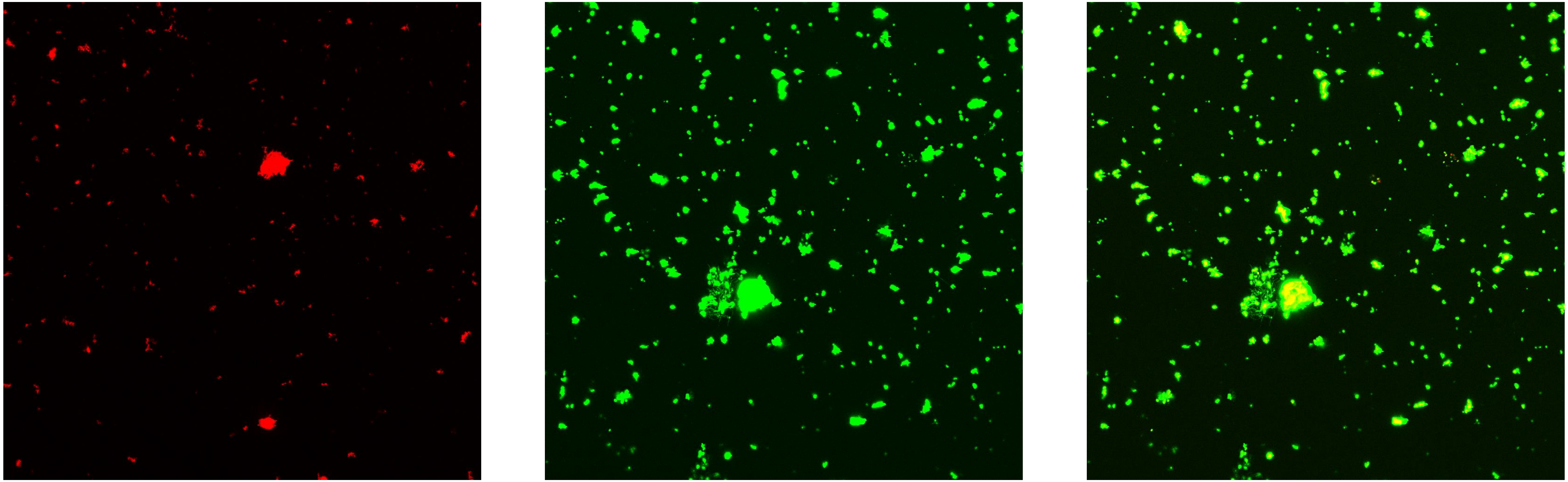

FGN PLT Merge

22I (Pre-treatment)

NO IIa

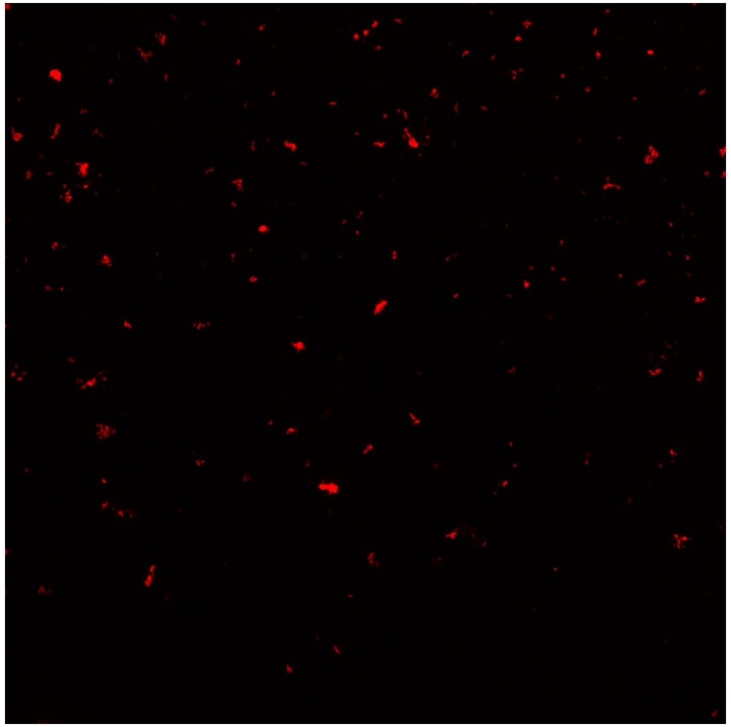

FGN

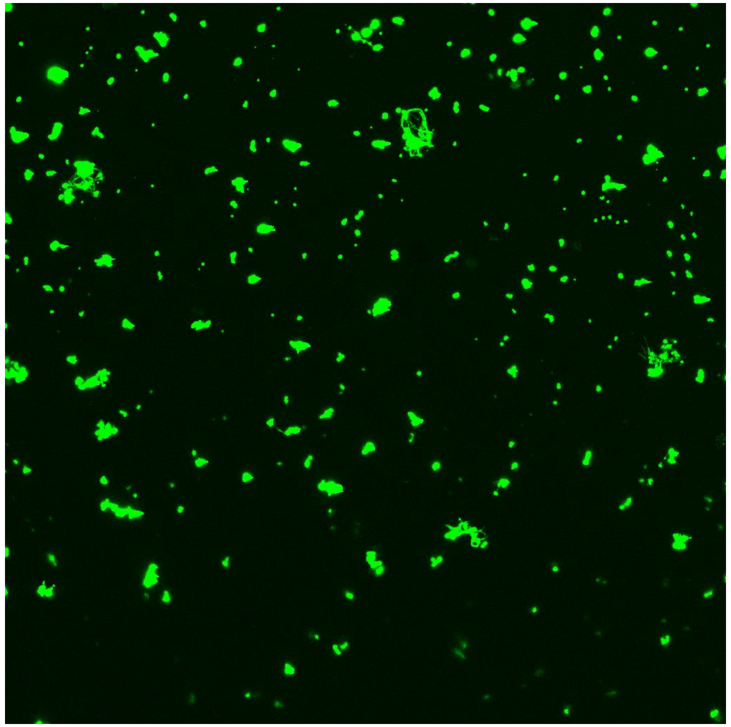

PLT

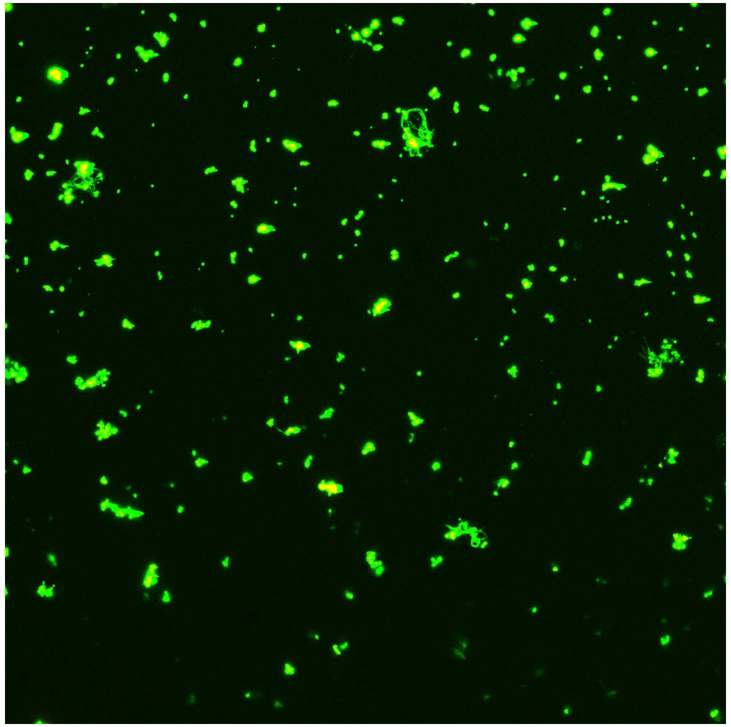

Merge

22II (Post-treatment)

NO IIa

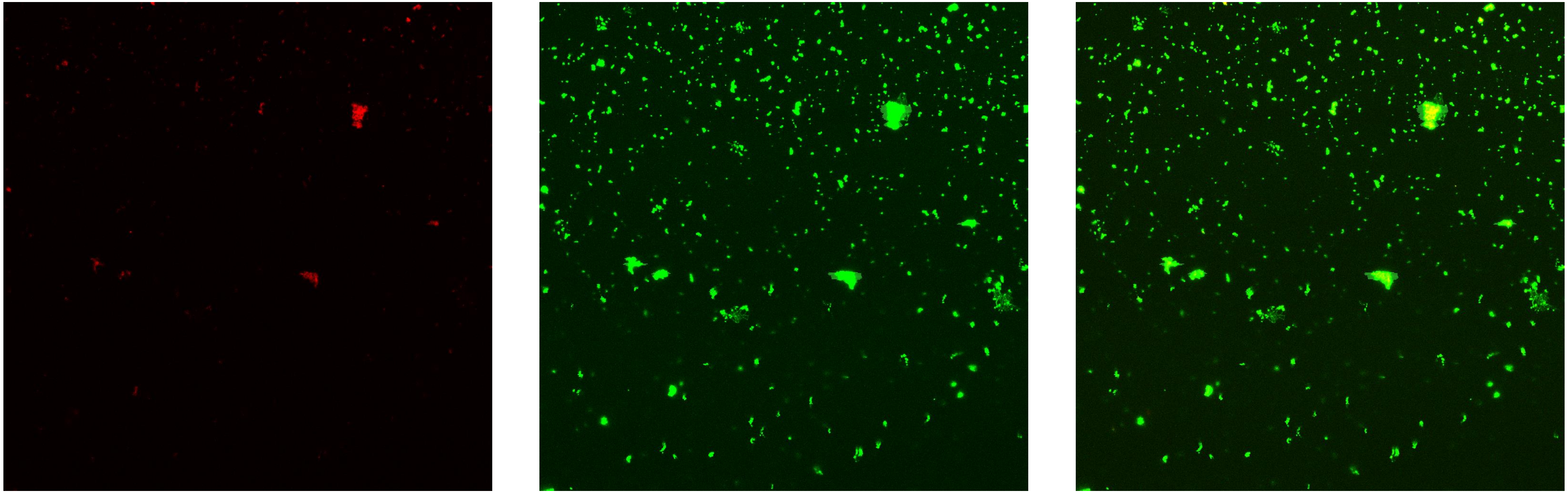

FGN PLT Merge

22II (Post-treatment)

NO IIa

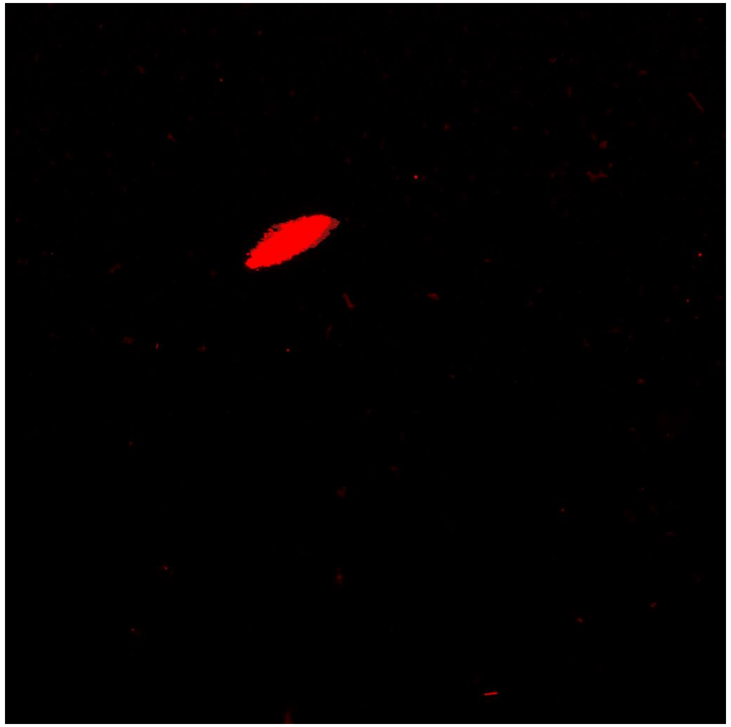

FGN

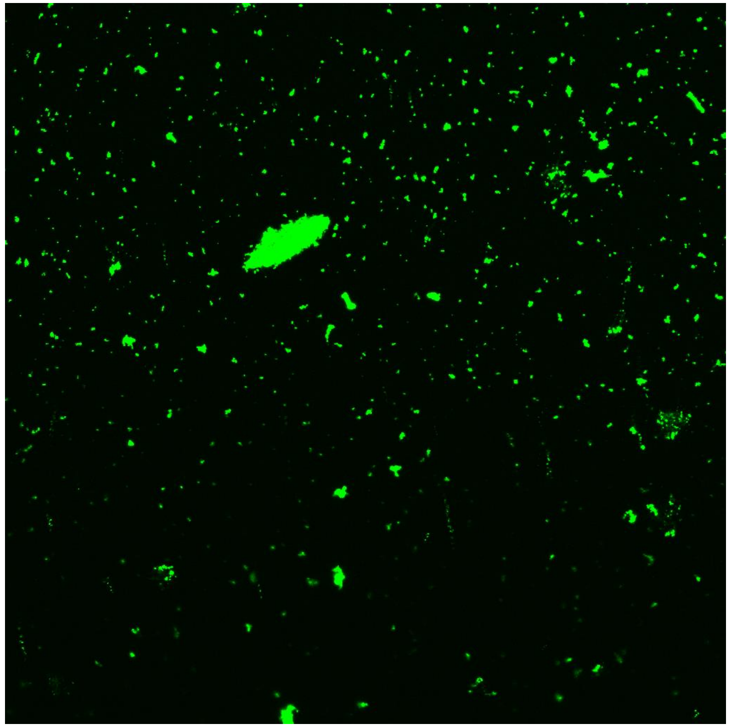

PLT

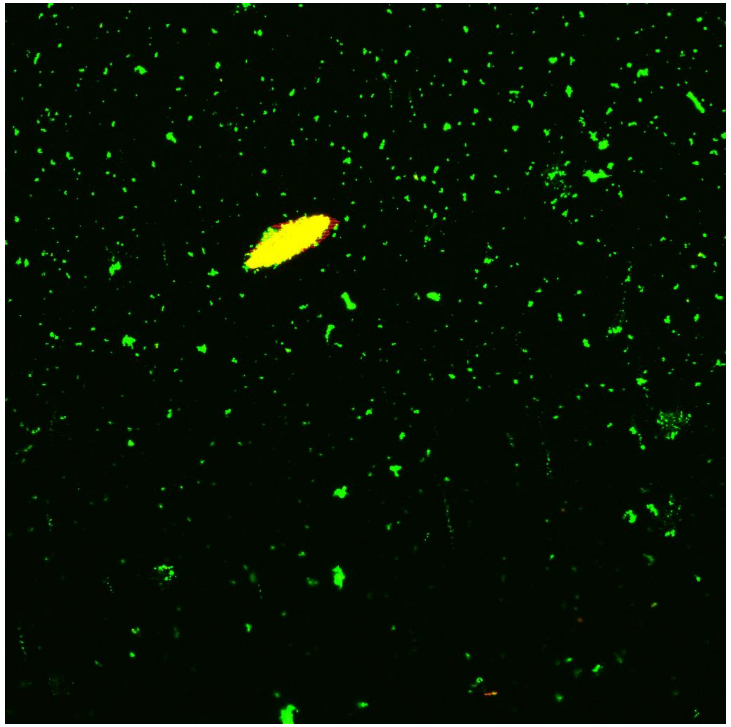

Merge

22II (Post-treatment)

SEM Images

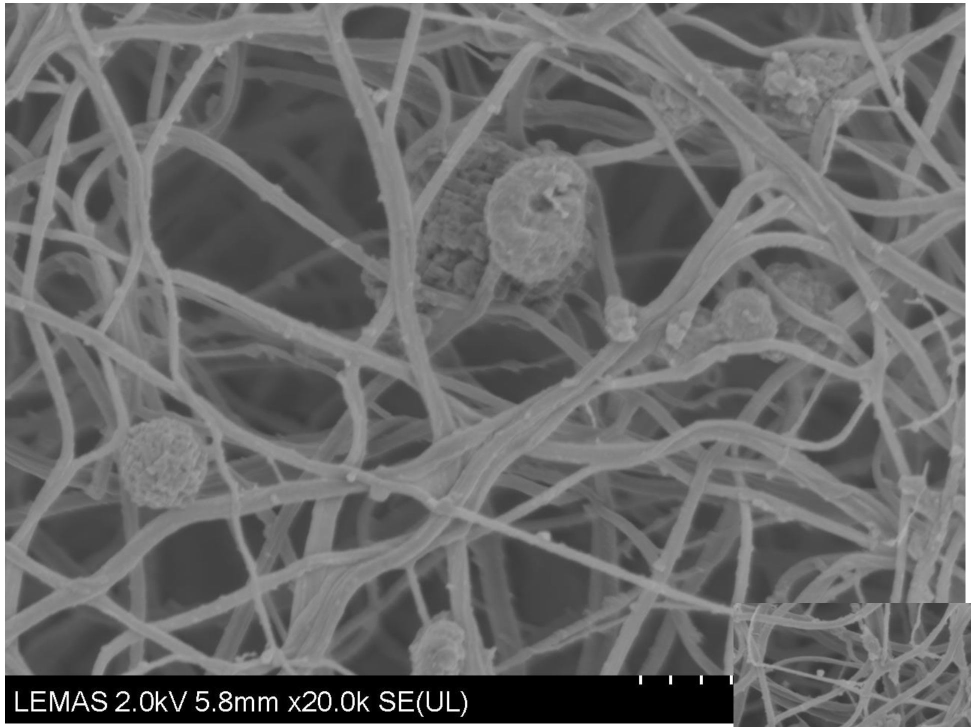

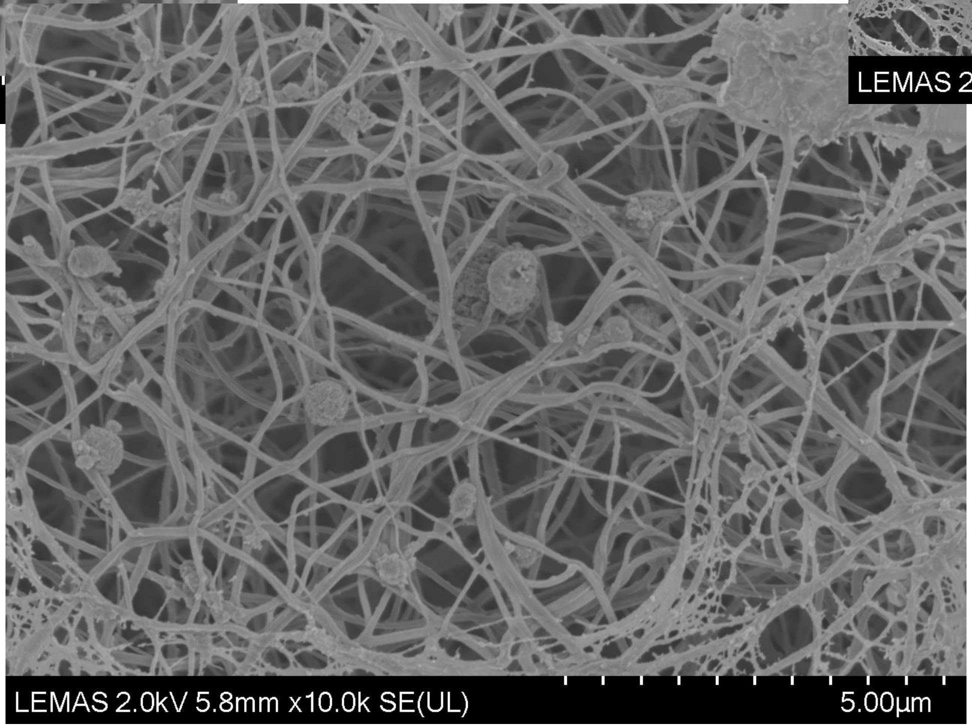

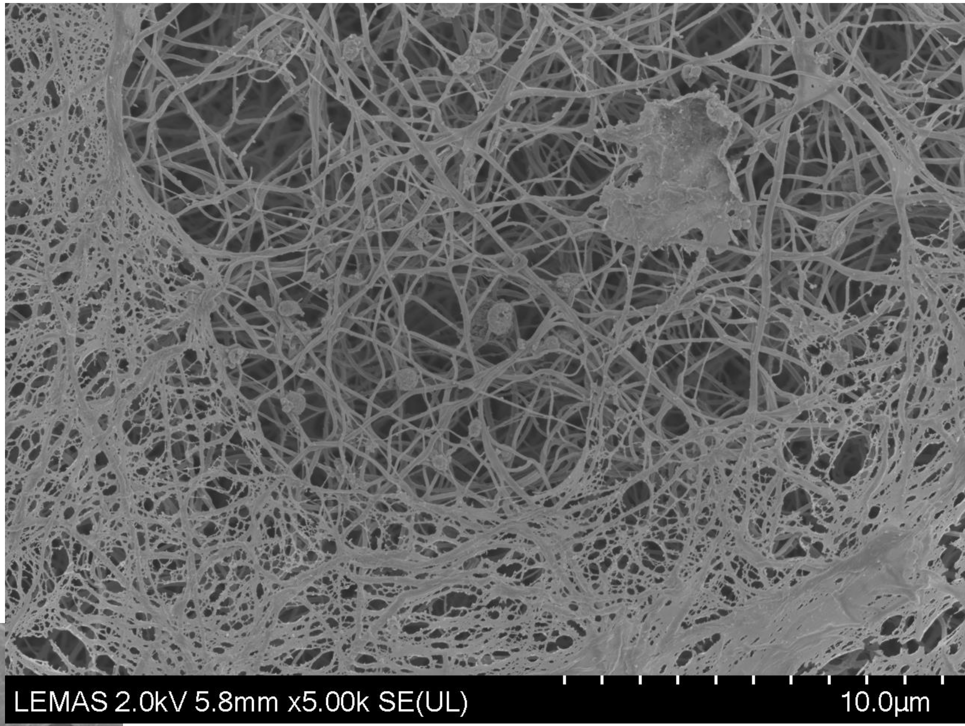

22I (Pre-treatment)

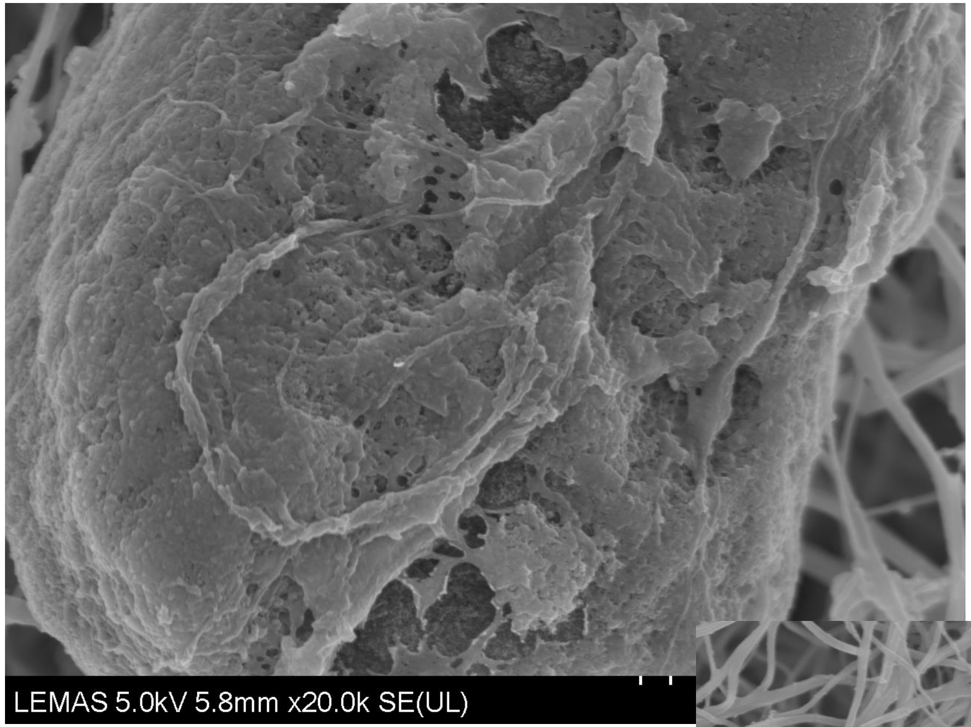

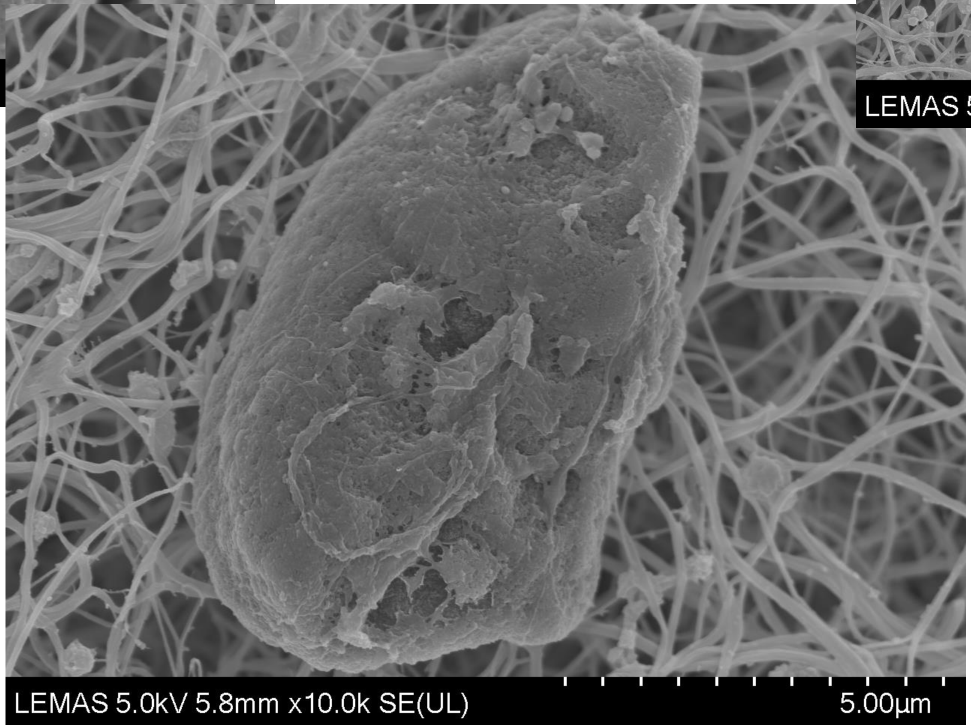

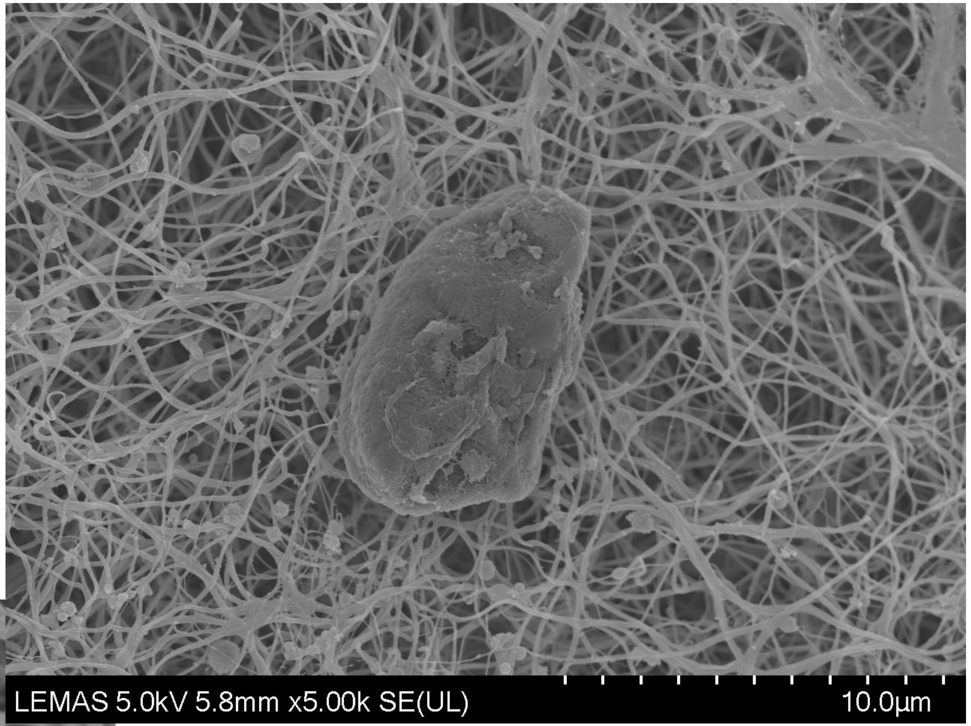

22I (Pre-treatment)

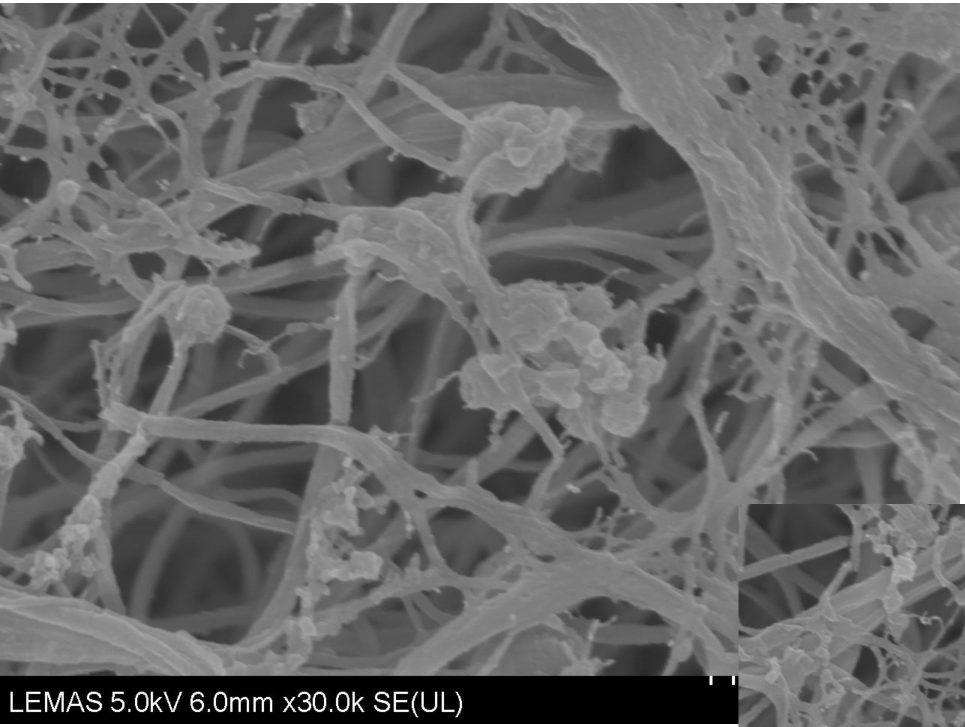

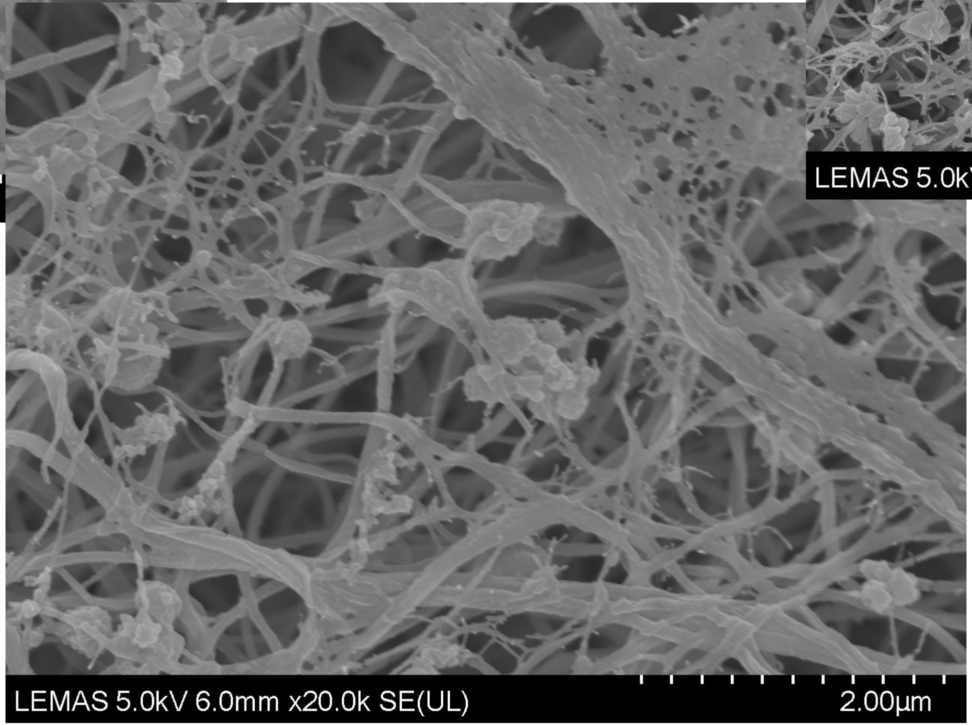

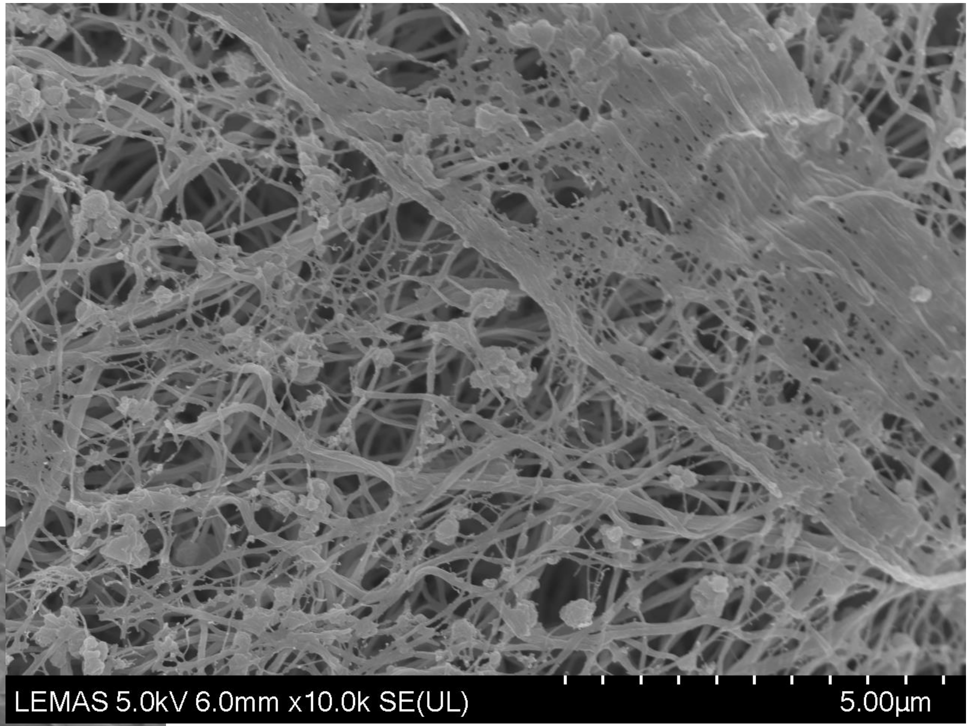

22II (Post-treatment)

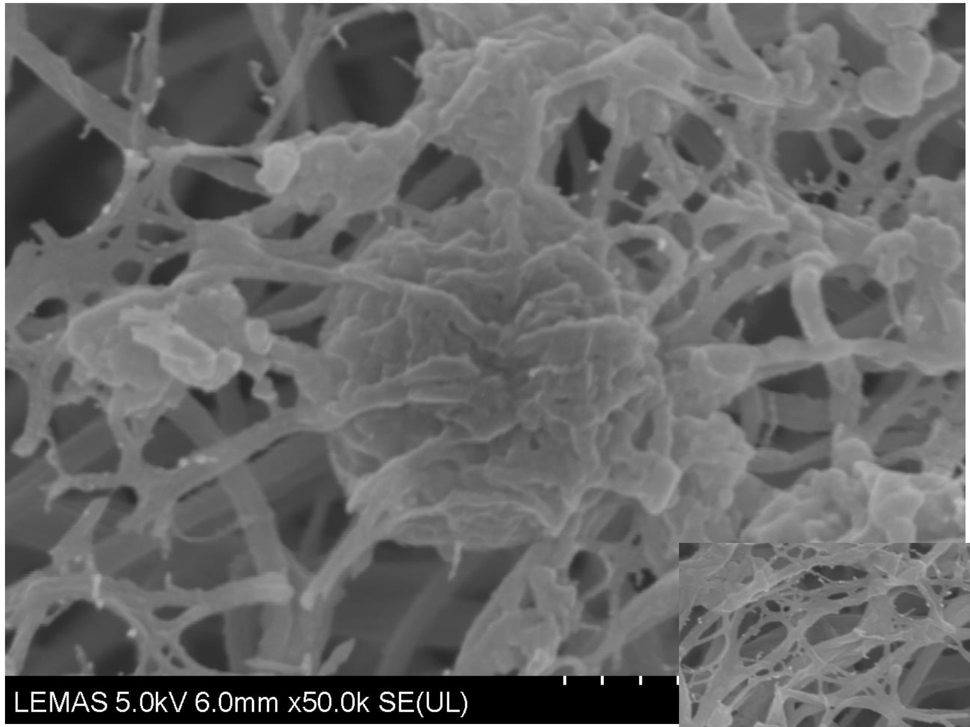

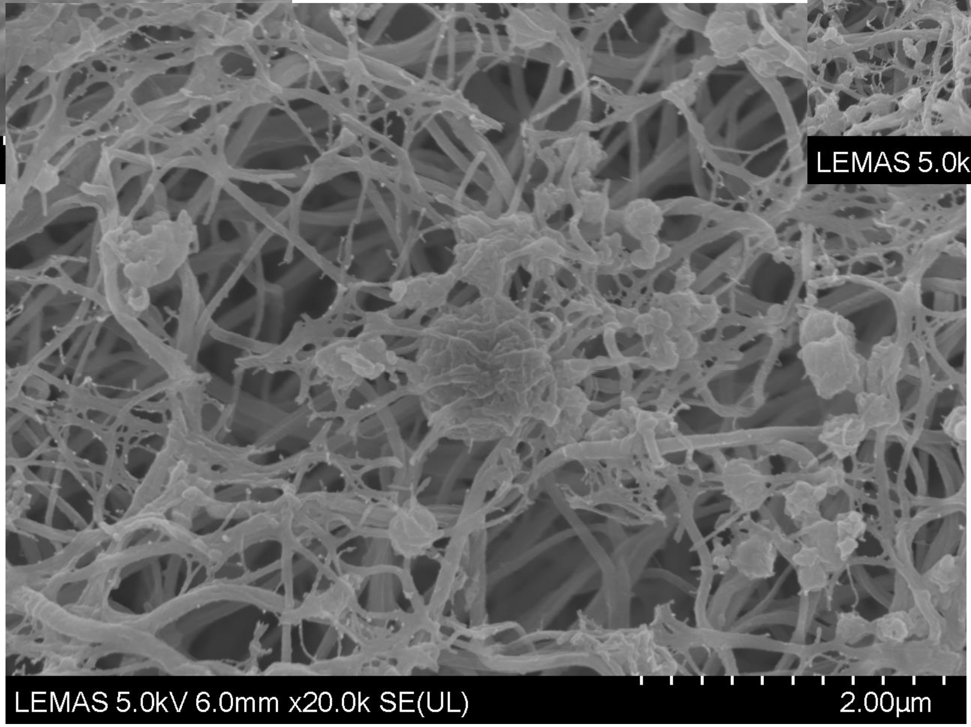

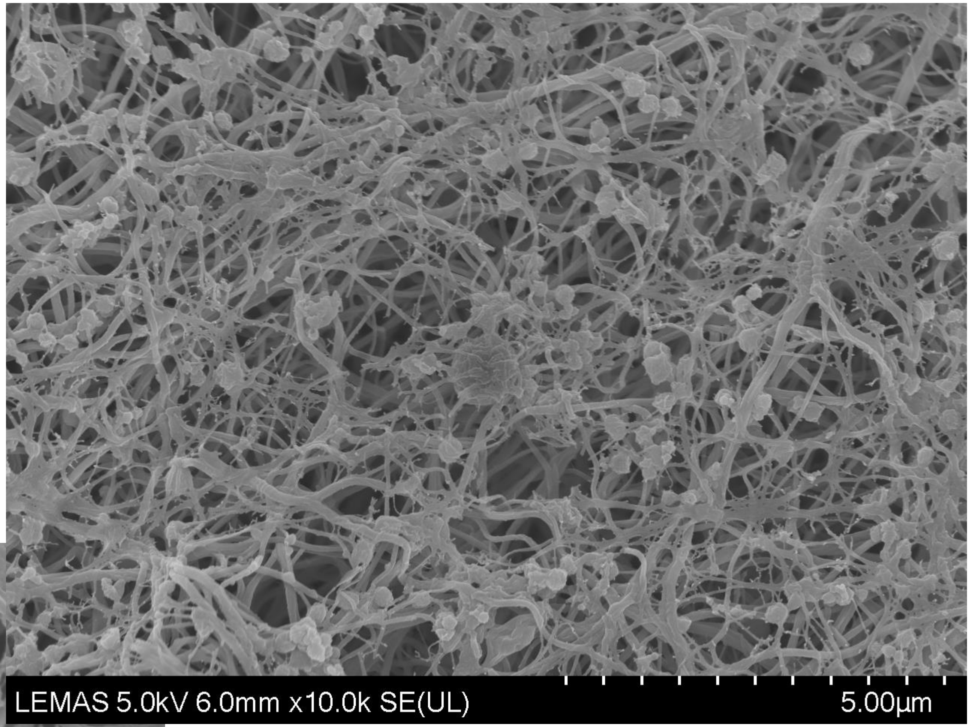

22II (Post-treatment)

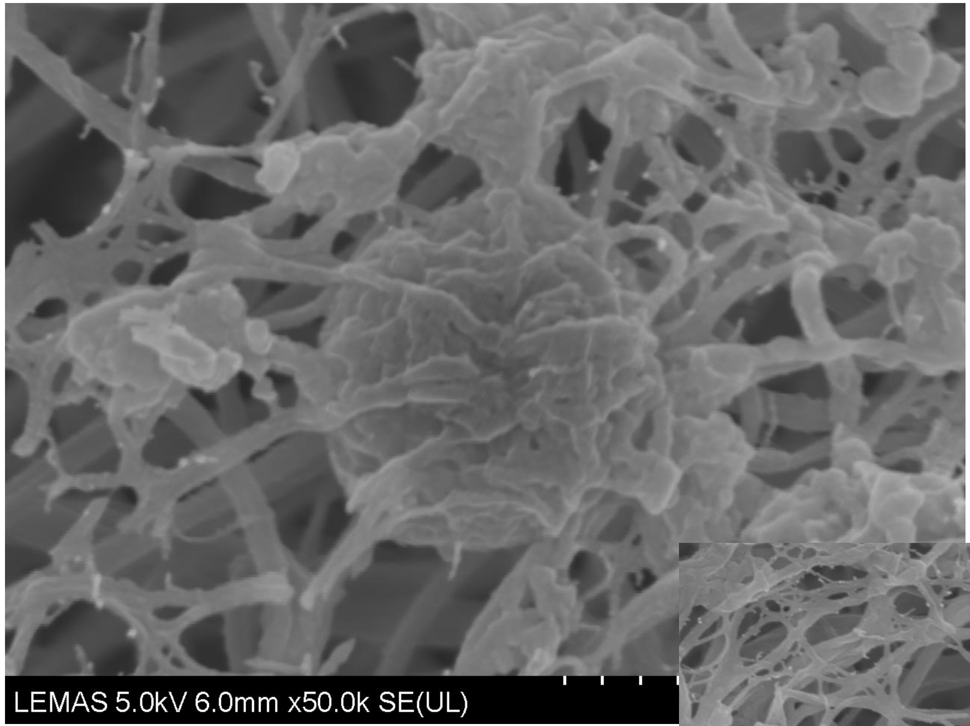

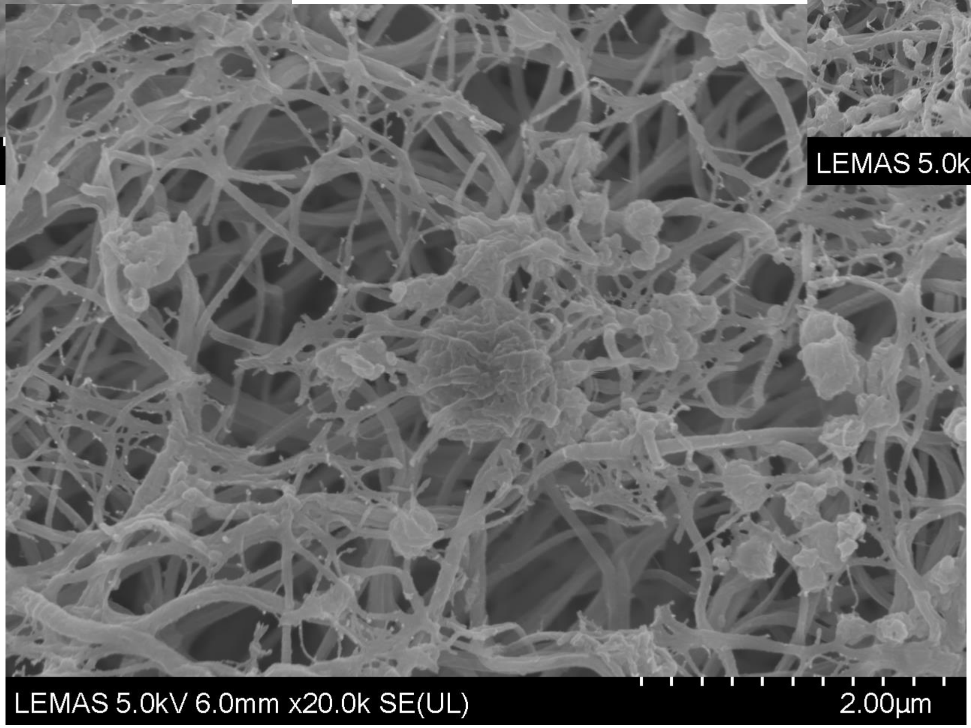

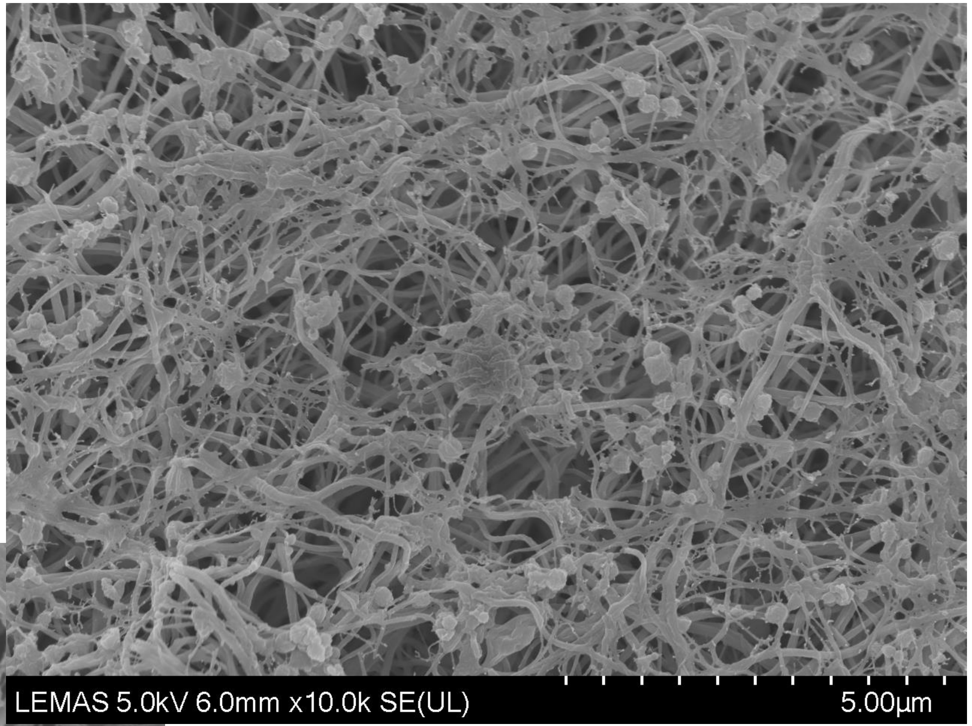

22II (Post-treatment)
