# Supplementary material for: Plasma from patients with pulmonary embolism show aggregates that reduce after anticoagulation
Source: Commun Med (Lond). 2023 Jan 28;3:12. doi: 10.1038/s43856-023-00242-8 (PMC9883810; doi:10.1038/s43856-023-00242-8)
Supplement: Supplementary file 2 — Supplementary Data 2 [file 43856_2023_242_MOESM2_ESM.pdf]

IBM SPSS Web Report - Microclot Cat. Variables Analysis.spv

Log

Log - Log - February 26, 2022

```
GET
  FILE='\\ds.leeds.ac.uk\staff\staff1\medrasa\VTE microclot clinical data.sav'.
DATASET NAME DataSet1 WINDOW=FRONT.
CROSSTABS
  /TABLES=Presenceofmicroclots BY SexM1F0 PreviousVTE DiagnosisPE1PEDVT0 VTEunprovoked SaddlePE
    BilateralPE CentralPE1peripheralPE0 SegmentaryPE1 SubsegmentaryPe1 Death1 CHD MI Stroke
    Hypertension HF DM Insulin Hyperchol HyperTG CKD COPD Asthma SuperficialVTE APS Autoimm.Disease IBD
    Betablocker ACEI ARB Calciumblocker ASA Statin Fenofibrate Aldosteroneantagonist Furosemide
    Metformin Amiodar IPP NSAIDS V55 FIIG20210A FVLeiden
  /FORMAT=AVALUE TABLES
  /STATISTICS=CHISQ
  /CELLS=COUNT
  /COUNT ROUND CELL.
```

Crosstabs

Crosstabs - Active Dataset - February 26, 2022

[DataSet1] \\ds.leeds.ac.uk\staff\staff1\medrasa\VTE microclot clinical data.sav

Crosstabs

Crosstabs - Warnings - February 26, 2022

Warnings

Warnings
The crosstabulation of Presence of microclots * V55 is empty.

IBM SPSS Web Report - Microclot Cat. Variables Analysis.spv

Crosstabs

Crosstabs - Case Processing Summary - February 26, 2022

IBM SPSS Web Report - Microclot Cat. Variables Analysis.spv

Case Processing Summary

	Cases					
	Valid		Missing		Total	
	N	Percent	N	Percent	N	Percent
Presence of microclots * Sex; M=1, F=0	35	100.0%	0	0.0%	35	100.0%
Presence of microclots * Previous VTE	35	100.0%	0	0.0%	35	100.0%
Presence of microclots * Diagnosis, PE=1, PE+DVT=0	35	100.0%	0	0.0%	35	100.0%
Presence of microclots * VTE unprovoked	35	100.0%	0	0.0%	35	100.0%
Presence of microclots * Saddle PE	35	100.0%	0	0.0%	35	100.0%
Presence of microclots * Bilateral PE	35	100.0%	0	0.0%	35	100.0%
Presence of microclots * Central PE=1, peripheral PE=0	35	100.0%	0	0.0%	35	100.0%
Presence of microclots * Segmentary PE=1	35	100.0%	0	0.0%	35	100.0%
Presence of microclots * Subsegmentary Pe=1	35	100.0%	0	0.0%	35	100.0%
Presence of microclots * Death=1	35	100.0%	0	0.0%	35	100.0%
Presence of microclots * CHD	35	100.0%	0	0.0%	35	100.0%
Presence of microclots * MI	35	100.0%	0	0.0%	35	100.0%
Presence of microclots * Stroke	35	100.0%	0	0.0%	35	100.0%
Presence of microclots * Hypertension	35	100.0%	0	0.0%	35	100.0%
Presence of microclots * HF	35	100.0%	0	0.0%	35	100.0%
Presence of microclots * DM	35	100.0%	0	0.0%	35	100.0%
Presence of microclots * Insulin	35	100.0%	0	0.0%	35	100.0%
Presence of microclots * Hyperchol	35	100.0%	0	0.0%	35	100.0%
Presence of microclots * HyperTG	35	100.0%	0	0.0%	35	100.0%
Presence of microclots * CKD	35	100.0%	0	0.0%	35	100.0%
Presence of microclots * COPD	35	100.0%	0	0.0%	35	100.0%
Presence of microclots * Asthma	35	100.0%	0	0.0%	35	100.0%
Presence of microclots * Superficial VTE	35	100.0%	0	0.0%	35	100.0%
Presence of microclots * APS	35	100.0%	0	0.0%	35	100.0%
Presence of microclots * Autoimm. Disease	35	100.0%	0	0.0%	35	100.0%
Presence of microclots * IBD	35	100.0%	0	0.0%	35	100.0%
Presence of microclots * Beta-blocker	35	100.0%	0	0.0%	35	100.0%
Presence of microclots * ACEI	35	100.0%	0	0.0%	35	100.0%
Presence of microclots * ARB	35	100.0%	0	0.0%	35	100.0%
Presence of microclots * Calcium blocker	35	100.0%	0	0.0%	35	100.0%
Presence of microclots * ASA	35	100.0%	0	0.0%	35	100.0%
Presence of microclots * Statin	35	100.0%	0	0.0%	35	100.0%
Presence of microclots * Fenofibrate	35	100.0%	0	0.0%	35	100.0%

IBM SPSS Web Report - Microclot Cat. Variables Analysis.spv

Metformin	35	100.0%	0	0.0%	35	100.0%
Presence of microclots * Amiodar.	35	100.0%	0	0.0%	35	100.0%
Presence of microclots * IPP	35	100.0%	0	0.0%	35	100.0%
Presence of microclots * NSAIDS	35	100.0%	0	0.0%	35	100.0%
Presence of microclots * FII G20210A	35	100.0%	0	0.0%	35	100.0%
Presence of microclots * FV Leiden	35	100.0%	0	0.0%	35	100.0%

Presence of microclots * Sex; M=1, F=0

Presence of microclots * Sex; M=1, F=0 - Crosstab - February 26, 2022

Crosstab

Count

		Sex; M=1, F=0		Total
		0	1	
Presence of microclots	0	19	8	27
	1	4	4	8
Total		23	12	35

Presence of microclots * Sex; M=1, F=0

Presence of microclots * Sex; M=1, F=0 - Chi-Square Tests - February 26, 2022

Chi-Square Tests

	Value	df	Asymptotic Significance (2-sided)	Exact Sig. (2-sided)	Exact Sig. (1-sided)
Pearson Chi-Square	1.137[a]	1	.286		
Continuity Correction[b]	.412	1	.521		
Likelihood Ratio	1.098	1	.295		
Fisher's Exact Test				.402	.257
Linear-by-Linear Association	1.104	1	.293		
N of Valid Cases	35				

a. 1 cells (25.0%) have expected count less than 5. The minimum expected count is 2.74.

b. Computed only for a 2x2 table

Presence of microclots * Previous VTE

Presence of microclots * Previous VTE - Crosstab - February 26, 2022

Crosstab

Count

		Previous VTE		Total
		0	1	
Presence of microclots	0	24	3	27
	1	7	1	8
Total		31	4	35

IBM SPSS Web Report - Microclot Cat. Variables Analysis.spv

Presence of microclots * Previous VTE

Presence of microclots * Previous VTE - Chi-Square Tests - February 26, 2022

Chi-Square Tests

	Value	df	Asymptotic Significance (2-sided)	Exact Sig. (2-sided)	Exact Sig. (1-sided)
Pearson Chi-Square	.012[a]	1	.914		
Continuity Correction[b]	.000	1	1.000		
Likelihood Ratio	.012	1	.914		
Fisher's Exact Test				1.000	.665
Linear-by-Linear Association	.011	1	.915		
N of Valid Cases	35				

a. 2 cells (50.0%) have expected count less than 5. The minimum expected count is .91.

b. Computed only for a 2x2 table

Presence of microclots * Diagnosis, PE=1, PE+DVT=0

Presence of microclots * Diagnosis, PE=1, PE+DVT=0 - Crosstab - February 26, 2022

Crosstab

Count

		Diagnosis, PE=1, PE+DVT=0		
		0	1	Total
Presence of microclots	0	8	19	27
	1	2	6	8
Total		10	25	35

Presence of microclots * Diagnosis, PE=1, PE+DVT=0

Presence of microclots * Diagnosis, PE=1, PE+DVT=0 - Chi-Square Tests - February 26, 2022

Chi-Square Tests

	Value	df	Asymptotic Significance (2-sided)	Exact Sig. (2-sided)	Exact Sig. (1-sided)
Pearson Chi-Square	.065[a]	1	.799		
Continuity Correction[b]	.000	1	1.000		
Likelihood Ratio	.066	1	.797		
Fisher's Exact Test				1.000	.589
Linear-by-Linear Association	.063	1	.802		
N of Valid Cases	35				

a. 1 cells (25.0%) have expected count less than 5. The minimum expected count is 2.29.

b. Computed only for a 2x2 table

IBM SPSS Web Report - Microclot Cat. Variables Analysis.spv

Presence of microclots * VTE unprovoked

Presence of microclots * VTE unprovoked - Crosstab - February 26, 2022

Crosstab

Count

		VTE unprovoked		Total
		0	1	
Presence of microclots	0	20	7	27
	1	5	3	8
Total		25	10	35

Presence of microclots * VTE unprovoked

Presence of microclots * VTE unprovoked - Chi-Square Tests - February 26, 2022

Chi-Square Tests

	Value	df	Asymptotic Significance (2-sided)	Exact Sig. (2-sided)	Exact Sig. (1-sided)
Pearson Chi-Square	.405[a]	1	.524		
Continuity Correction[b]	.036	1	.849		
Likelihood Ratio	.391	1	.532		
Fisher's Exact Test				.661	.411
Linear-by-Linear Association	.394	1	.530		
N of Valid Cases	35				

a. 1 cells (25.0%) have expected count less than 5. The minimum expected count is 2.29.

b. Computed only for a 2x2 table

Presence of microclots * Saddle PE

Presence of microclots * Saddle PE - Crosstab - February 26, 2022

Crosstab

Count

		Saddle PE		Total
		0	1	
Presence of microclots	0	24	3	27
	1	7	1	8
Total		31	4	35

IBM SPSS Web Report - Microclot Cat. Variables Analysis.spv

Presence of microclots * Saddle PE

Presence of microclots * Saddle PE - Chi-Square Tests - February 26, 2022

Chi-Square Tests

	Value	df	Asymptotic Significance (2-sided)	Exact Sig. (2-sided)	Exact Sig. (1-sided)
Pearson Chi-Square	.012[a]	1	.914		
Continuity Correction[b]	.000	1	1.000		
Likelihood Ratio	.012	1	.914		
Fisher's Exact Test				1.000	.665
Linear-by-Linear Association	.011	1	.915		
N of Valid Cases	35				

a. 2 cells (50.0%) have expected count less than 5. The minimum expected count is .91.

b. Computed only for a 2x2 table

Presence of microclots * Bilateral PE

Presence of microclots * Bilateral PE - Crosstab - February 26, 2022

Crosstab

Count

		Bilateral PE		Total
		0	1	
Presence of microclots	0	9	18	27
	1	1	7	8
Total		10	25	35

Presence of microclots * Bilateral PE

Presence of microclots * Bilateral PE - Chi-Square Tests - February 26, 2022

Chi-Square Tests

	Value	df	Asymptotic Significance (2-sided)	Exact Sig. (2-sided)	Exact Sig. (1-sided)
Pearson Chi-Square	1.312[a]	1	.252		
Continuity Correction[b]	.490	1	.484		
Likelihood Ratio	1.479	1	.224		
Fisher's Exact Test				.390	.250
Linear-by-Linear Association	1.275	1	.259		
N of Valid Cases	35				

a. 1 cells (25.0%) have expected count less than 5. The minimum expected count is 2.29.

b. Computed only for a 2x2 table

IBM SPSS Web Report - Microclot Cat. Variables Analysis.spv

Presence of microclots * Central PE=1, peripheral PE=0

Presence of microclots * Central PE=1, peripheral PE=0 - Crosstab - February 26, 2022

Crosstab

Count

		Central PE=1, peripheral PE=0		Total
		0	1	
Presence of microclots	0	12	15	27
	1	4	4	8
Total		16	19	35

Presence of microclots * Central PE=1, peripheral PE=0

Presence of microclots * Central PE=1, peripheral PE=0 - Chi-Square Tests - February 26, 2022

Chi-Square Tests

	Value	df	Asymptotic Significance (2-sided)	Exact Sig. (2-sided)	Exact Sig. (1-sided)
Pearson Chi-Square	.077[a]	1	.782		
Continuity Correction[b]	.000	1	1.000		
Likelihood Ratio	.077	1	.782		
Fisher's Exact Test				1.000	.548
Linear-by-Linear Association	.075	1	.785		
N of Valid Cases	35				

a. 2 cells (50.0%) have expected count less than 5. The minimum expected count is 3.66.

b. Computed only for a 2x2 table

Presence of microclots * Segmentary PE=1

Presence of microclots * Segmentary PE=1 - Crosstab - February 26, 2022

Crosstab

Count

		Segmentary PE=1		Total
		0	1	
Presence of microclots	0	16	11	27
	1	4	4	8
Total		20	15	35

IBM SPSS Web Report - Microclot Cat. Variables Analysis.spv

Presence of microclots * Segmentary PE=1

Presence of microclots * Segmentary PE=1 - Chi-Square Tests - February 26, 2022

Chi-Square Tests

	Value	df	Asymptotic Significance (2-sided)	Exact Sig. (2-sided)	Exact Sig. (1-sided)
Pearson Chi-Square	.216[a]	1	.642		
Continuity Correction[b]	.003	1	.954		
Likelihood Ratio	.215	1	.643		
Fisher's Exact Test				.700	.473
Linear-by-Linear Association	.210	1	.647		
N of Valid Cases	35				

a. 2 cells (50.0%) have expected count less than 5. The minimum expected count is 3.43.

b. Computed only for a 2x2 table

Presence of microclots * Subsegmentary Pe=1

Presence of microclots * Subsegmentary Pe=1 - Crosstab - February 26, 2022

Crosstab

Count

		Subsegmentary Pe=1		Total
		0	1	
Presence of microclots	0	21	6	27
	1	7	1	8
Total		28	7	35

Presence of microclots * Subsegmentary Pe=1

Presence of microclots * Subsegmentary Pe=1 - Chi-Square Tests - February 26, 2022

Chi-Square Tests

	Value	df	Asymptotic Significance (2-sided)	Exact Sig. (2-sided)	Exact Sig. (1-sided)
Pearson Chi-Square	.365[a]	1	.546		
Continuity Correction[b]	.010	1	.920		
Likelihood Ratio	.396	1	.529		
Fisher's Exact Test				1.000	.484
Linear-by-Linear Association	.354	1	.552		
N of Valid Cases	35				

a. 1 cells (25.0%) have expected count less than 5. The minimum expected count is 1.60.

b. Computed only for a 2x2 table

IBM SPSS Web Report - Microclot Cat. Variables Analysis.spv

Presence of microclots * Death=1

Presence of microclots * Death=1 - Crosstab - February 26, 2022

Crosstab

Count

		Death=1		Total
		0	1	
Presence of microclots	0	25	2	27
	1	8	0	8
Total		33	2	35

Presence of microclots * Death=1

Presence of microclots * Death=1 - Chi-Square Tests - February 26, 2022

Chi-Square Tests

	Value	df	Asymptotic Significance (2-sided)	Exact Sig. (2-sided)	Exact Sig. (1-sided)
Pearson Chi-Square	.629[a]	1	.428		
Continuity Correction[b]	.000	1	1.000		
Likelihood Ratio	1.073	1	.300		
Fisher's Exact Test				1.000	.590
Linear-by-Linear Association	.611	1	.435		
N of Valid Cases	35				

a. 2 cells (50.0%) have expected count less than 5. The minimum expected count is .46.

b. Computed only for a 2x2 table

Presence of microclots * CHD

Presence of microclots * CHD - Crosstab - February 26, 2022

Crosstab

Count

		CHD		Total
		0	1	
Presence of microclots	0	21	6	27
	1	6	2	8
Total		27	8	35

IBM SPSS Web Report - Microclot Cat. Variables Analysis.spv

Presence of microclots * CHD

Presence of microclots * CHD - Chi-Square Tests - February 26, 2022

Chi-Square Tests

	Value	df	Asymptotic Significance (2-sided)	Exact Sig. (2-sided)	Exact Sig. (1-sided)
Pearson Chi-Square	.027[a]	1	.869		
Continuity Correction[b]	.000	1	1.000		
Likelihood Ratio	.027	1	.870		
Fisher's Exact Test				1.000	.604
Linear-by-Linear Association	.026	1	.871		
N of Valid Cases	35				

a. 1 cells (25.0%) have expected count less than 5. The minimum expected count is 1.83.

b. Computed only for a 2x2 table

Presence of microclots * MI

Presence of microclots * MI - Crosstab - February 26, 2022

Crosstab

Count

		MI		Total
		0	1	
Presence of microclots	0	25	2	27
	1	7	1	8
Total		32	3	35

Presence of microclots * MI

Presence of microclots * MI - Chi-Square Tests - February 26, 2022

Chi-Square Tests

	Value	df	Asymptotic Significance (2-sided)	Exact Sig. (2-sided)	Exact Sig. (1-sided)
Pearson Chi-Square	.204[a]	1	.651		
Continuity Correction[b]	.000	1	1.000		
Likelihood Ratio	.188	1	.664		
Fisher's Exact Test				.553	.553
Linear-by-Linear Association	.198	1	.656		
N of Valid Cases	35				

a. 2 cells (50.0%) have expected count less than 5. The minimum expected count is .69.

b. Computed only for a 2x2 table

IBM SPSS Web Report - Microclot Cat. Variables Analysis.spv

Presence of microclots * Stroke

Presence of microclots * Stroke - Crosstab - February 26, 2022

Crosstab

Count

		Stroke		
		0	1	Total
Presence of microclots	0	26	1	27
	1	8	0	8
Total		34	1	35

Presence of microclots * Stroke

Presence of microclots * Stroke - Chi-Square Tests - February 26, 2022

Chi-Square Tests

	Value	df	Asymptotic Significance (2-sided)	Exact Sig. (2-sided)	Exact Sig. (1-sided)
Pearson Chi-Square	.305[a]	1	.581		
Continuity Correction[b]	.000	1	1.000		
Likelihood Ratio	.528	1	.468		
Fisher's Exact Test				1.000	.771
Linear-by-Linear Association	.296	1	.586		
N of Valid Cases	35				

a. 2 cells (50.0%) have expected count less than 5. The minimum expected count is .23.

b. Computed only for a 2x2 table

Presence of microclots * Hypertension

Presence of microclots * Hypertension - Crosstab - February 26, 2022

Crosstab

Count

		Hypertension		
		0	1	Total
Presence of microclots	0	13	14	27
	1	2	6	8
Total		15	20	35

IBM SPSS Web Report - Microclot Cat. Variables Analysis.spv

Presence of microclots * Hypertension

Presence of microclots * Hypertension - Chi-Square Tests - February 26, 2022

Chi-Square Tests

	Value	df	Asymptotic Significance (2-sided)	Exact Sig. (2-sided)	Exact Sig. (1-sided)
Pearson Chi-Square	1.350[a]	1	.245		
Continuity Correction[b]	.571	1	.450		
Likelihood Ratio	1.413	1	.235		
Fisher's Exact Test				.419	.228
Linear-by-Linear Association	1.312	1	.252		
N of Valid Cases	35				

a. 2 cells (50.0%) have expected count less than 5. The minimum expected count is 3.43.

b. Computed only for a 2x2 table

Presence of microclots * HF

Presence of microclots * HF - Crosstab - February 26, 2022

Crosstab

Count

		HF		Total
		0	1	
Presence of microclots	0	25	2	27
	1	8	0	8
Total		33	2	35

Presence of microclots * HF

Presence of microclots * HF - Chi-Square Tests - February 26, 2022

Chi-Square Tests

	Value	df	Asymptotic Significance (2-sided)	Exact Sig. (2-sided)	Exact Sig. (1-sided)
Pearson Chi-Square	.629[a]	1	.428		
Continuity Correction[b]	.000	1	1.000		
Likelihood Ratio	1.073	1	.300		
Fisher's Exact Test				1.000	.590
Linear-by-Linear Association	.611	1	.435		
N of Valid Cases	35				

a. 2 cells (50.0%) have expected count less than 5. The minimum expected count is .46.

b. Computed only for a 2x2 table

IBM SPSS Web Report - Microclot Cat. Variables Analysis.spv

Presence of microclots * DM

Presence of microclots * DM - Crosstab - February 26, 2022

Crosstab

Count

		DM		
		0	1	Total
Presence of microclots	0	22	5	27
	1	8	0	8
Total		30	5	35

Presence of microclots * DM

Presence of microclots * DM - Chi-Square Tests - February 26, 2022

Chi-Square Tests

	Value	df	Asymptotic Significance (2-sided)	Exact Sig. (2-sided)	Exact Sig. (1-sided)
Pearson Chi-Square	1.728[a]	1	.189		
Continuity Correction[b]	.547	1	.460		
Likelihood Ratio	2.833	1	.092		
Fisher's Exact Test				.315	.249
Linear-by-Linear Association	1.679	1	.195		
N of Valid Cases	35				

a. 2 cells (50.0%) have expected count less than 5. The minimum expected count is 1.14.

b. Computed only for a 2x2 table

Presence of microclots * Insulin

Presence of microclots * Insulin - Crosstab - February 26, 2022

Crosstab

Count

		Insulin		
		0	1	Total
Presence of microclots	0	26	1	27
	1	8	0	8
Total		34	1	35

IBM SPSS Web Report - Microclot Cat. Variables Analysis.spv

Presence of microclots * Insulin

Presence of microclots * Insulin - Chi-Square Tests - February 26, 2022

Chi-Square Tests

	Value	df	Asymptotic Significance (2-sided)	Exact Sig. (2-sided)	Exact Sig. (1-sided)
Pearson Chi-Square	.305[a]	1	.581		
Continuity Correction[b]	.000	1	1.000		
Likelihood Ratio	.528	1	.468		
Fisher's Exact Test				1.000	.771
Linear-by-Linear Association	.296	1	.586		
N of Valid Cases	35				

a. 2 cells (50.0%) have expected count less than 5. The minimum expected count is .23.

b. Computed only for a 2x2 table

Presence of microclots * Hyperchol

Presence of microclots * Hyperchol - Crosstab - February 26, 2022

Crosstab

Count

		Hyperchol		Total
		0	1	
Presence of microclots	0	18	9	27
	1	4	4	8
Total		22	13	35

Presence of microclots * Hyperchol

Presence of microclots * Hyperchol - Chi-Square Tests - February 26, 2022

Chi-Square Tests

	Value	df	Asymptotic Significance (2-sided)	Exact Sig. (2-sided)	Exact Sig. (1-sided)
Pearson Chi-Square	.734[a]	1	.392		
Continuity Correction[b]	.194	1	.660		
Likelihood Ratio	.718	1	.397		
Fisher's Exact Test				.433	.325
Linear-by-Linear Association	.713	1	.398		
N of Valid Cases	35				

a. 1 cells (25.0%) have expected count less than 5. The minimum expected count is 2.97.

b. Computed only for a 2x2 table

IBM SPSS Web Report - Microclot Cat. Variables Analysis.spv

Presence of microclots * HyperTG

Presence of microclots * HyperTG - Crosstab - February 26, 2022

Crosstab

Count

		HyperTG		Total
		0	1	
Presence of microclots	0	24	3	27
	1	7	1	8
Total		31	4	35

Presence of microclots * HyperTG

Presence of microclots * HyperTG - Chi-Square Tests - February 26, 2022

Chi-Square Tests

	Value	df	Asymptotic Significance (2-sided)	Exact Sig. (2-sided)	Exact Sig. (1-sided)
Pearson Chi-Square	.012[a]	1	.914		
Continuity Correction[b]	.000	1	1.000		
Likelihood Ratio	.012	1	.914		
Fisher's Exact Test				1.000	.665
Linear-by-Linear Association	.011	1	.915		
N of Valid Cases	35				

a. 2 cells (50.0%) have expected count less than 5. The minimum expected count is .91.

b. Computed only for a 2x2 table

Presence of microclots * CKD

Presence of microclots * CKD - Crosstab - February 26, 2022

Crosstab

Count

		CKD		Total
		0	1	
Presence of microclots	0	22	5	27
	1	8	0	8
Total		30	5	35

IBM SPSS Web Report - Microclot Cat. Variables Analysis.spv

Presence of microclots * CKD

Presence of microclots * CKD - Chi-Square Tests - February 26, 2022

Chi-Square Tests

	Value	df	Asymptotic Significance (2-sided)	Exact Sig. (2-sided)	Exact Sig. (1-sided)
Pearson Chi-Square	1.728[a]	1	.189		
Continuity Correction[b]	.547	1	.460		
Likelihood Ratio	2.833	1	.092		
Fisher's Exact Test				.315	.249
Linear-by-Linear Association	1.679	1	.195		
N of Valid Cases	35				

a. 2 cells (50.0%) have expected count less than 5. The minimum expected count is 1.14.

b. Computed only for a 2x2 table

Presence of microclots * COPD

Presence of microclots * COPD - Crosstab - February 26, 2022

Crosstab

Count

		COPD		Total
		0	1	
Presence of microclots	0	26	1	27
	1	8	0	8
Total		34	1	35

Presence of microclots * COPD

Presence of microclots * COPD - Chi-Square Tests - February 26, 2022

Chi-Square Tests

	Value	df	Asymptotic Significance (2-sided)	Exact Sig. (2-sided)	Exact Sig. (1-sided)
Pearson Chi-Square	.305[a]	1	.581		
Continuity Correction[b]	.000	1	1.000		
Likelihood Ratio	.528	1	.468		
Fisher's Exact Test				1.000	.771
Linear-by-Linear Association	.296	1	.586		
N of Valid Cases	35				

a. 2 cells (50.0%) have expected count less than 5. The minimum expected count is .23.

b. Computed only for a 2x2 table

IBM SPSS Web Report - Microclot Cat. Variables Analysis.spv

Presence of microclots * Asthma

Presence of microclots * Asthma - Crosstab - February 26, 2022

Crosstab

Count

		Asthma		Total
		0	1	
Presence of microclots	0	25	2	27
	1	8	0	8
Total		33	2	35

Presence of microclots * Asthma

Presence of microclots * Asthma - Chi-Square Tests - February 26, 2022

Chi-Square Tests

	Value	df	Asymptotic Significance (2-sided)	Exact Sig. (2-sided)	Exact Sig. (1-sided)
Pearson Chi-Square	.629[a]	1	.428		
Continuity Correction[b]	.000	1	1.000		
Likelihood Ratio	1.073	1	.300		
Fisher's Exact Test				1.000	.590
Linear-by-Linear Association	.611	1	.435		
N of Valid Cases	35				

a. 2 cells (50.0%) have expected count less than 5. The minimum expected count is .46.

b. Computed only for a 2x2 table

Presence of microclots * Superficial VTE

Presence of microclots * Superficial VTE - Crosstab - February 26, 2022

Crosstab

Count

		Superficial VTE		Total
		0	1	
Presence of microclots	0	25	2	27
	1	8	0	8
Total		33	2	35

IBM SPSS Web Report - Microclot Cat. Variables Analysis.spv

Presence of microclots * Superficial VTE

Presence of microclots * Superficial VTE - Chi-Square Tests - February 26, 2022

Chi-Square Tests

	Value	df	Asymptotic Significance (2-sided)	Exact Sig. (2-sided)	Exact Sig. (1-sided)
Pearson Chi-Square	.629[a]	1	.428		
Continuity Correction[b]	.000	1	1.000		
Likelihood Ratio	1.073	1	.300		
Fisher's Exact Test				1.000	.590
Linear-by-Linear Association	.611	1	.435		
N of Valid Cases	35				

a. 2 cells (50.0%) have expected count less than 5. The minimum expected count is .46.

b. Computed only for a 2x2 table

Presence of microclots * APS

Presence of microclots * APS - Crosstab - February 26, 2022

Crosstab

Count

		APS	
		0	Total
Presence of microclots	0	27	27
	1	8	8
Total		35	35

Presence of microclots * APS

Presence of microclots * APS - Chi-Square Tests - February 26, 2022

Chi-Square Tests

	Value
Pearson Chi-Square	.[a]
N of Valid Cases	35

a. No statistics are computed because APS is a constant.

Presence of microclots * Autoimm. Disease

Presence of microclots * Autoimm. Disease - Crosstab - February 26, 2022

Crosstab

Count

		Autoimm. Disease		
		0	1	Total
Presence of microclots	0	26	1	27
	1	8	0	8
Total		34	1	35

IBM SPSS Web Report - Microclot Cat. Variables Analysis.spv

Presence of microclots * Autoimm. Disease

Presence of microclots * Autoimm. Disease - Chi-Square Tests - February 26, 2022

Chi-Square Tests

	Value	df	Asymptotic Significance (2-sided)	Exact Sig. (2-sided)	Exact Sig. (1-sided)
Pearson Chi-Square	.305[a]	1	.581		
Continuity Correction[b]	.000	1	1.000		
Likelihood Ratio	.528	1	.468		
Fisher's Exact Test				1.000	.771
Linear-by-Linear Association	.296	1	.586		
N of Valid Cases	35				

a. 2 cells (50.0%) have expected count less than 5. The minimum expected count is .23.

b. Computed only for a 2x2 table

Presence of microclots * IBD

Presence of microclots * IBD - Crosstab - February 26, 2022

Crosstab

Count

		IBD		Total
		0	1	
Presence of microclots	0	26	1	27
	1	8	0	8
Total		34	1	35

Presence of microclots * IBD

Presence of microclots * IBD - Chi-Square Tests - February 26, 2022

Chi-Square Tests

	Value	df	Asymptotic Significance (2-sided)	Exact Sig. (2-sided)	Exact Sig. (1-sided)
Pearson Chi-Square	.305[a]	1	.581		
Continuity Correction[b]	.000	1	1.000		
Likelihood Ratio	.528	1	.468		
Fisher's Exact Test				1.000	.771
Linear-by-Linear Association	.296	1	.586		
N of Valid Cases	35				

a. 2 cells (50.0%) have expected count less than 5. The minimum expected count is .23.

b. Computed only for a 2x2 table

IBM SPSS Web Report - Microclot Cat. Variables Analysis.spv

Presence of microclots * Beta-blocker

Presence of microclots * Beta-blocker - Crosstab - February 26, 2022

Crosstab

Count

		Beta-blocker		Total
		0	1	
Presence of microclots	0	14	13	27
	1	5	3	8
Total		19	16	35

Presence of microclots * Beta-blocker

Presence of microclots * Beta-blocker - Chi-Square Tests - February 26, 2022

Chi-Square Tests

	Value	df	Asymptotic Significance (2-sided)	Exact Sig. (2-sided)	Exact Sig. (1-sided)
Pearson Chi-Square	.282[a]	1	.595		
Continuity Correction[b]	.016	1	.899		
Likelihood Ratio	.285	1	.593		
Fisher's Exact Test				.700	.452
Linear-by-Linear Association	.274	1	.601		
N of Valid Cases	35				

a. 2 cells (50.0%) have expected count less than 5. The minimum expected count is 3.66.

b. Computed only for a 2x2 table

Presence of microclots * ACEI

Presence of microclots * ACEI - Crosstab - February 26, 2022

Crosstab

Count

		ACEI		Total
		0	1	
Presence of microclots	0	14	13	27
	1	2	6	8
Total		16	19	35

IBM SPSS Web Report - Microclot Cat. Variables Analysis.spv

Presence of microclots * ACEI

Presence of microclots * ACEI - Chi-Square Tests - February 26, 2022

Chi-Square Tests

	Value	df	Asymptotic Significance (2-sided)	Exact Sig. (2-sided)	Exact Sig. (1-sided)
Pearson Chi-Square	1.793[a]	1	.181		
Continuity Correction[b]	.874	1	.350		
Likelihood Ratio	1.873	1	.171		
Fisher's Exact Test				.244	.176
Linear-by-Linear Association	1.742	1	.187		
N of Valid Cases	35				

a. 2 cells (50.0%) have expected count less than 5. The minimum expected count is 3.66.

b. Computed only for a 2x2 table

Presence of microclots * ARB

Presence of microclots * ARB - Crosstab - February 26, 2022

Crosstab

Count

		ARB	
		0	Total
Presence of microclots	0	27	27
	1	8	8
Total		35	35

Presence of microclots * ARB

Presence of microclots * ARB - Chi-Square Tests - February 26, 2022

Chi-Square Tests

	Value
Pearson Chi-Square	.[a]
N of Valid Cases	35

a. No statistics are computed because ARB is a constant.

Presence of microclots * Calcium blocker

Presence of microclots * Calcium blocker - Crosstab - February 26, 2022

Crosstab

Count

		Calcium blocker		
		0	1	Total
Presence of microclots	0	26	1	27
	1	8	0	8
Total		34	1	35

IBM SPSS Web Report - Microclot Cat. Variables Analysis.spv

Presence of microclots * Calcium blocker

Presence of microclots * Calcium blocker - Chi-Square Tests - February 26, 2022

Chi-Square Tests

	Value	df	Asymptotic Significance (2-sided)	Exact Sig. (2-sided)	Exact Sig. (1-sided)
Pearson Chi-Square	.305[a]	1	.581		
Continuity Correction[b]	.000	1	1.000		
Likelihood Ratio	.528	1	.468		
Fisher's Exact Test				1.000	.771
Linear-by-Linear Association	.296	1	.586		
N of Valid Cases	35				

a. 2 cells (50.0%) have expected count less than 5. The minimum expected count is .23.

b. Computed only for a 2x2 table

Presence of microclots * ASA

Presence of microclots * ASA - Crosstab - February 26, 2022

Crosstab

Count

		ASA		Total
		0	1	
Presence of microclots	0	18	9	27
	1	6	2	8
Total		24	11	35

Presence of microclots * ASA

Presence of microclots * ASA - Chi-Square Tests - February 26, 2022

Chi-Square Tests

	Value	df	Asymptotic Significance (2-sided)	Exact Sig. (2-sided)	Exact Sig. (1-sided)
Pearson Chi-Square	.199[a]	1	.656		
Continuity Correction[b]	.000	1	.990		
Likelihood Ratio	.205	1	.651		
Fisher's Exact Test				1.000	.508
Linear-by-Linear Association	.193	1	.660		
N of Valid Cases	35				

a. 1 cells (25.0%) have expected count less than 5. The minimum expected count is 2.51.

b. Computed only for a 2x2 table

IBM SPSS Web Report - Microclot Cat. Variables Analysis.spv

Presence of microclots * Statin

Presence of microclots * Statin - Crosstab - February 26, 2022

Crosstab

Count

		Statin		Total
		0	1	
Presence of microclots	0	18	9	27
	1	6	2	8
Total		24	11	35

Presence of microclots * Statin

Presence of microclots * Statin - Chi-Square Tests - February 26, 2022

Chi-Square Tests

	Value	df	Asymptotic Significance (2-sided)	Exact Sig. (2-sided)	Exact Sig. (1-sided)
Pearson Chi-Square	.199[a]	1	.656		
Continuity Correction[b]	.000	1	.990		
Likelihood Ratio	.205	1	.651		
Fisher's Exact Test				1.000	.508
Linear-by-Linear Association	.193	1	.660		
N of Valid Cases	35				

a. 1 cells (25.0%) have expected count less than 5. The minimum expected count is 2.51.

b. Computed only for a 2x2 table

Presence of microclots * Fenofibrate

Presence of microclots * Fenofibrate - Crosstab - February 26, 2022

Crosstab

Count

		Fenofibrate	Total
		0	
Presence of microclots	0	27	27
	1	8	8
Total		35	35

Presence of microclots * Fenofibrate

Presence of microclots * Fenofibrate - Chi-Square Tests - February 26, 2022

Chi-Square Tests

	Value
Pearson Chi-Square	.[a]
N of Valid Cases	35

a. No statistics are computed because Fenofibrate is a constant.

IBM SPSS Web Report - Microclot Cat. Variables Analysis.spv

Presence of microclots * Aldosterone antagonist

Presence of microclots * Aldosterone antagonist - Crosstab - February 26, 2022

Crosstab

Count

		Aldosterone antagonist	Total
		0	
Presence of microclots	0	27	27
	1	8	8
Total		35	35

Presence of microclots * Aldosterone antagonist

Presence of microclots * Aldosterone antagonist - Chi-Square Tests - February 26, 2022

Chi-Square Tests

	Value
Pearson Chi-Square	.[a]
N of Valid Cases	35

a. No statistics are computed because Aldosterone antagonist is a constant.

Presence of microclots * Furosemide

Presence of microclots * Furosemide - Crosstab - February 26, 2022

Crosstab

Count

		Furosemide	Total
		0	
Presence of microclots	0	27	27
	1	8	8
Total		35	35

Presence of microclots * Furosemide

Presence of microclots * Furosemide - Chi-Square Tests - February 26, 2022

Chi-Square Tests

	Value
Pearson Chi-Square	.[a]
N of Valid Cases	35

a. No statistics are computed because Furosemide is a constant.

Presence of microclots * Metformin

Presence of microclots * Metformin - Crosstab - February 26, 2022

Crosstab

Count

		Metformin		Total
		0	1	
Presence of microclots	0	25	2	27
	1	8	0	8
Total		33	2	35

IBM SPSS Web Report - Microclot Cat. Variables Analysis.spv

Presence of microclots * Metformin

Presence of microclots * Metformin - Chi-Square Tests - February 26, 2022

Chi-Square Tests

	Value	df	Asymptotic Significance (2-sided)	Exact Sig. (2-sided)	Exact Sig. (1-sided)
Pearson Chi-Square	.629[a]	1	.428		
Continuity Correction[b]	.000	1	1.000		
Likelihood Ratio	1.073	1	.300		
Fisher's Exact Test				1.000	.590
Linear-by-Linear Association	.611	1	.435		
N of Valid Cases	35				

a. 2 cells (50.0%) have expected count less than 5. The minimum expected count is .46.

b. Computed only for a 2x2 table

Presence of microclots * Amiodar.

Presence of microclots * Amiodar. - Crosstab - February 26, 2022

Crosstab

Count

		Amiodar.	
		0	Total
Presence of microclots	0	27	27
	1	8	8
Total		35	35

Presence of microclots * Amiodar.

Presence of microclots * Amiodar. - Chi-Square Tests - February 26, 2022

Chi-Square Tests

	Value
Pearson Chi-Square	.[a]
N of Valid Cases	35

a. No statistics are computed because Amiodar. is a constant.

Presence of microclots * IPP

Presence of microclots * IPP - Crosstab - February 26, 2022

Crosstab

Count

		IPP		
		0	1	Total
Presence of microclots	0	21	6	27
	1	7	1	8
Total		28	7	35

IBM SPSS Web Report - Microclot Cat. Variables Analysis.spv

Presence of microclots * IPP

Presence of microclots * IPP - Chi-Square Tests - February 26, 2022

Chi-Square Tests

	Value	df	Asymptotic Significance (2-sided)	Exact Sig. (2-sided)	Exact Sig. (1-sided)
Pearson Chi-Square	.365[a]	1	.546		
Continuity Correction[b]	.010	1	.920		
Likelihood Ratio	.396	1	.529		
Fisher's Exact Test				1.000	.484
Linear-by-Linear Association	.354	1	.552		
N of Valid Cases	35				

a. 1 cells (25.0%) have expected count less than 5. The minimum expected count is 1.60.

b. Computed only for a 2x2 table

Presence of microclots * NSAIDS

Presence of microclots * NSAIDS - Crosstab - February 26, 2022

Crosstab

Count

		NSAIDS		Total
		0	1	
Presence of microclots	0	25	2	27
	1	8	0	8
Total		33	2	35

Presence of microclots * NSAIDS

Presence of microclots * NSAIDS - Chi-Square Tests - February 26, 2022

Chi-Square Tests

	Value	df	Asymptotic Significance (2-sided)	Exact Sig. (2-sided)	Exact Sig. (1-sided)
Pearson Chi-Square	.629[a]	1	.428		
Continuity Correction[b]	.000	1	1.000		
Likelihood Ratio	1.073	1	.300		
Fisher's Exact Test				1.000	.590
Linear-by-Linear Association	.611	1	.435		
N of Valid Cases	35				

a. 2 cells (50.0%) have expected count less than 5. The minimum expected count is .46.

b. Computed only for a 2x2 table

IBM SPSS Web Report - Microclot Cat. Variables Analysis.spv

Presence of microclots * FII G20210A

Presence of microclots * FII G20210A - Crosstab - February 26, 2022

Crosstab

Count

		FII G20210A	
		GG	Total
Presence of microclots	0	27	27
	1	8	8
Total		35	35

Presence of microclots * FII G20210A

Presence of microclots * FII G20210A - Chi-Square Tests - February 26, 2022

Chi-Square Tests

	Value
Pearson Chi-Square	.[a]
N of Valid Cases	35

a. No statistics are computed because FII G20210A is a constant.

Presence of microclots * FV Leiden

Presence of microclots * FV Leiden - Crosstab - February 26, 2022

Crosstab

Count

		FV Leiden		
		GA	GG	Total
Presence of microclots	0	2	25	27
	1	0	8	8
Total		2	33	35

Presence of microclots * FV Leiden

Presence of microclots * FV Leiden - Chi-Square Tests - February 26, 2022

Chi-Square Tests

	Value	df	Asymptotic Significance (2-sided)	Exact Sig. (2-sided)	Exact Sig. (1-sided)
Pearson Chi-Square	.629[a]	1	.428		
Continuity Correction[b]	.000	1	1.000		
Likelihood Ratio	1.073	1	.300		
Fisher's Exact Test				1.000	.590
N of Valid Cases	35				

a. 2 cells (50.0%) have expected count less than 5. The minimum expected count is .46.

b. Computed only for a 2x2 table
