# Supplementary material for: Plasma from patients with pulmonary embolism show aggregates that reduce after anticoagulation
Source: Commun Med (Lond). 2023 Jan 28;3:12. doi: 10.1038/s43856-023-00242-8 (PMC9883810; doi:10.1038/s43856-023-00242-8)
Supplement: Supplementary file 3 — Supplementary Data 3 [file 43856_2023_242_MOESM3_ESM.pdf]

IBM SPSS Web Report - Microclot Cont. Variables Analysis.spv

Log

Log - Log - February 26, 2022

```
GET DATA
  /TYPE=XLSX
  /FILE='\\ds.leeds.ac.uk\staff\staff1\medrasa\PE-plasma_Leeds_01.08.2018_12.04.2019_7.07mod.2021_25.2.2022.xlsx'
  /SHEET=name 'Arkusz2'
  /CELLRANGE=FULL
  /READNAMES=ON
  /DATATYPEMIN PERCENTAGE=95.0
  /HIDDEN IGNORE=YES.
EXECUTE.
DATASET NAME DataSet1 WINDOW=FRONT.

SAVE OUTFILE='\\ds.leeds.ac.uk\staff\staff1\medrasa\VTE microclot clinical data.sav'
  /COMPRESSED.
T-TEST GROUPS=Presenceofmicroclots(0 1)
  /MISSING=ANALYSIS
  /VARIABLES=Agey BMIkgm2 DayssincePEsymptoms Glucosemmoll WBC103ul NEUT103ul LYM103ul MONO103ul
    EOS103ul BASO103ul RBC106ul HGBgdl HCT PLT103ul CHOLmgdl LDLmgdl HDLmgdl TGmgdl Fibrinogengl CLTmin
    Ks109cm2 AntiXaUI Lagtime_min ETP_nM•min Peak_nM ttPeak_min PAIIngml tPAAgngml TAFIact TAFIAg a2AP
    Plasminogen Lpamgdl proBNPpgml TnTpgml CRPmgl Ddimerngml Pselektynangml PF4ngml HcyuM AT PC PS
    FVIII B2GPIIgG B2GPIIgM aCLIgG aCLIgM MaxOD LagTime TimetoMaxOD AverageRateofClotting
    MaximumRateofClotting Fibers100um @2MaxOD deltaLysis deltaOD
  /CRITERIA=CI(.95).
```

T-Test

T-Test - Active Dataset - February 26, 2022

[DataSet1] \\ds.leeds.ac.uk\staff\staff1\medrasa\VTE microclot clinical data.sav

IBM SPSS Web Report - Microclot Cont. Variables Analysis.spv

T-Test

T-Test - Group Statistics - February 26, 2022

IBM SPSS Web Report - Microclot Cont. Variables Analysis.spv

Group Statistics

	Presence of microclots	N	Mean	Std. Deviation	Std. Error Mean
Age, y	0	27	61.63	16.068	3.092
	1	8	68.25	14.469	5.116
BMI kg/m2	0	27	27.920194180933430	6.136309823624813	1.180933376166910
	1	8	29.910613988605757	3.888380134216765	1.374749980367866
Days since PE symptoms	0	27	6.81	5.137	.989
	1	8	4.13	3.137	1.109
Glucose, mmol/l	0	27	7.0059	2.83451	.54550
	1	8	6.9775	2.60059	.91945
WBC 10^3/ul	0	27	10.6578	3.45996	.66587
	1	8	9.8662	2.59712	.91822
NEUT 10^3/ul	0	27	7.0152	2.74032	.52737
	1	8	6.4600	2.07462	.73349
LYM 10^3/ul	0	27	2.4159	1.46713	.28235
	1	8	2.3137	.66973	.23679
MONO 10^3/ul	0	27	.8363	.33965	.06537
	1	8	.7000	.16630	.05880
EOS 10^3/ul	0	27	.1600	.13185	.02537
	1	8	.2438	.20500	.07248
BASO 10^3/ul	0	27	.05863	.055331	.010648
	1	8	.08750	.070862	.025054
RBC10^6/ul	0	27	4.4619	.63182	.12159
	1	8	4.4750	.54324	.19207
HGB g/dl	0	27	13.581	1.5140	.2914
	1	8	13.550	1.5892	.5619
HCT %	0	27	40.270	4.7821	.9203
	1	8	39.888	4.2448	1.5008
PLT 10^3/ul	0	27	257.33	99.919	19.229
	1	8	191.38	46.898	16.581
CHOL, mg/dl	0	27	188.00	45.843	8.822
	1	8	175.00	48.937	17.302
LDL, mg/dl	0	27	115.07	40.006	7.699
	1	8	111.75	50.307	17.786
HDL, mg/dl	0	27	48.674	23.1790	4.4608
	1	8	46.875	10.8554	3.8380
TG, mg/dl	0	27	165.56	122.453	23.566
	1	8	101.13	40.123	14.186
Fibrinogen, g/l	0	27	3.6341	1.38846	.26721
	1	8	4.0000	1.01905	.36029
CLT, min	0	27	126.519753086419750	30.722692355614193	5.912584900581304
	1	8	110.500000000000000	24.075773240809050	8.512071260442850
Ks, 10^-9cm^2	0	27	5.261092298335735	2.536739928199604	.488195826803371
	1	8	4.832663309473533	2.509343743234187	.887186988584464
Anti-Xa, UI	0	27	.0259	.05308	.01022
	1	8	.0250	.03162	.01118
Lagtime_(min)	0	27	4.3981	2.08325	.40092
	1	8	5.1700	1.81451	.64152
ETP_(nM•min)	0	27	1918.5922	538.34421	103.60439
	1	8	1419.9287	423.29313	149.65672
Peak_(nM)	0	27	304.5719	137.20830	26.40575
	1	8	208.2600	113.13591	39.99958
ttPeak_(min)	0	27	7.8641	2.72742	.52489
	1	8	9.7000	3.36433	1.18947
PAI-I, ng/ml	0	27	25.808267	16.8441680	3.2416616
	1	8	17.836375	12.4405317	4.3983922
tPA Ag, ng/ml	0	27	11.128904	5.3735507	1.0341403
	1	8	11.035775	6.0928636	2.1541526
TAFI act, %	0	27	95.04263	9.245621	1.779321
	1	8	92.24463	7.732943	2.734008
TAFI Ag, %	0	27	81.900459	19.5257596	3.7577342
	1	8	78.661500	22.8533974	8.0798961
a2AP, %	0	27	100.41	13.681	2.633
	1	8	96.25	15.691	5.548
Plasminogen, %	0	27	111.00	15.601	3.002
	1	8	102.75	8.120	2.871
Lp(a), mg/dl	0	27	22.111	30.8559	5.9382
	1	8	17.963	27.9437	9.8796
proBNP, pg/ml	0	27	2118.33	4940.685	950.835
	1	8	2228.88	2685.012	949.295
TnT, pg/ml	0	27	30.233	38.8222	7.4713
	1	8	68.313	78.5253	27.7629
CRP, mg/l	0	27	32.6363	33.89771	6.52362
	1	8	32.1725	46.91446	16.58677
D-dimer, ng/ml	0	27	5939.3222	6359.54647	1223.89529

IBM SPSS Web Report - Microclot Cont. Variables Analysis.spv

	1	8	64.4213	16.42025	5.80543
Hcy, uM	0	27	27.096	32.1578	6.1888
	1	8	17.363	5.7351	2.0277
AT, %	0	27	95.00	13.837	2.663
	1	8	97.88	14.506	5.128
PC, %	0	27	122.26	26.460	5.092
	1	8	125.38	27.349	9.669
PS, %	0	27	107.41	22.747	4.378
	1	8	93.88	20.594	7.281
FVIII, %	0	27	172.63	49.654	9.556
	1	8	155.63	40.602	14.355
B2GPI IgG	0	27	.837	1.1439	.2201
	1	8	4.725	6.5027	2.2990
B2GPI IgM	0	27	8.019	22.0517	4.2439
	1	8	3.313	4.8316	1.7082
aCL IgG	0	27	4.148	3.3523	.6452
	1	8	4.712	2.8048	.9917
aCL IgM	0	27	9.263	12.1791	2.3439
	1	8	5.763	3.2222	1.1392
Max OD	0	27	.46611	.220340	.042404
	1	8	.53125	.102306	.036171
Lag Time	0	27	228.37	93.612	18.016
	1	8	281.00	304.134	107.528
Time to Max OD	0	27	5629.70	2236.564	430.427
	1	8	6474.50	3252.218	1149.833
Average Rate of Clotting	0	27	.00065378	.000412581	.000079401
	1	8	.00057900	.000292333	.000103355
Maximum Rate of Clotting	0	27	.00133041	.000733755	.000141211
	1	8	.00133913	.000686538	.000242728
Fibers / 100 um	0	25	21.2408	4.38615	.87723
	1	8	23.1488	3.51037	1.24110
2Max OD	0	27	.54744	.217733	.041903
	1	8	.52863	.152369	.053871
deltaLysis	0	25	115.20	2571.178	514.236
	1	6	278.00	987.197	403.022
delta OD	0	27	-.081333333333333	.201930681175496	.038861577711439
	1	8	.002625000000000	.178863026204012	.063237629366202

IBM SPSS Web Report - Microclot Cont. Variables Analysis.spv

T-Test

T-Test - Independent Samples Test - February 26, 2022

IBM SPSS Web Report - Microclot Cont. Variables Analysis.spv

Independent Samples Test

		Levene's Test for Equality of Variances		t-test for Equality of Means						
									95% Confidence Interval of the D	
		F	Sig.	t	df	Sig. (2-tailed)	Mean Difference	Std. Error Difference	Lower	Up
Age, y	Equal variances assumed	.119	.733	-1.045	33	.304	-6.620	6.337	-19.513	
	Equal variances not assumed			-1.108	12.597	.289	-6.620	5.978	-19.576	
BMI kg/m2	Equal variances assumed	2.446	.127	-.862	33	.395	-1.990419807672328	2.307993992414361	-6.686068891659964	2.70522
	Equal variances not assumed			-1.098	18.439	.286	-1.990419807672328	1.812330308598967	-5.791494839382574	1.81065
Days since PE symptoms	Equal variances assumed	1.485	.232	1.397	33	.172	2.690	1.925	-1.227	
	Equal variances not assumed			1.811	19.269	.086	2.690	1.486	-.417	
Glucose, mmol/l	Equal variances assumed	.158	.693	.025	33	.980	.02843	1.12169	-2.25366	
	Equal variances not assumed			.027	12.382	.979	.02843	1.06909	-2.29297	
WBC 10^3/ul	Equal variances assumed	.742	.395	.597	33	.555	.79153	1.32671	-1.90769	
	Equal variances not assumed			.698	15.169	.496	.79153	1.13425	-1.62372	
NEUT 10^3/ul	Equal variances assumed	.613	.439	.528	33	.601	.55519	1.05196	-1.58504	
	Equal variances not assumed			.615	15.027	.548	.55519	.90340	-1.37006	
LYM 10^3/ul	Equal variances assumed	.744	.395	.190	33	.851	.10218	.53872	-.99385	
	Equal variances not assumed			.277	26.587	.784	.10218	.36850	-.65446	
MONO 10^3/ul	Equal variances assumed	2.796	.104	1.089	33	.284	.13630	.12521	-.11845	
	Equal variances not assumed			1.550	24.797	.134	.13630	.08792	-.04485	
EOS 10^3/ul	Equal variances assumed	2.547	.120	-1.384	33	.176	-.08375	.06053	-.20690	
	Equal variances not assumed			-1.091	8.786	.304	-.08375	.07679	-.25812	
BASO 10^3/ul	Equal variances assumed	1.570	.219	-1.216	33	.233	-.028870	.023737	-.077163	
	Equal variances not assumed			-1.061	9.673	.315	-.028870	.027223	-.089805	
RBC10^6/ul	Equal variances assumed	.206	.653	-.053	33	.958	-.01315	.24720	-.51608	
	Equal variances not assumed			-.058	13.166	.955	-.01315	.22732	-.50361	
HGB g/dl	Equal variances assumed	.055	.817	.051	33	.960	.0315	.6160	-1.2218	
	Equal variances not assumed			.050	11.056	.961	.0315	.6329	-1.3608	
HCT %	Equal variances assumed	.193	.664	.204	33	.840	.3829	1.8812	-3.4444	
	Equal variances not assumed			.217	12.768	.831	.3829	1.7605	-3.4274	
PLT 10^3/ul	Equal variances assumed	2.159	.151	1.795	33	.082	65.958	36.745	-8.800	
	Equal variances not assumed			2.598	25.885	.015	65.958	25.391	13.755	
CHOL, mg/dl	Equal variances assumed	.011	.918	.694	33	.492	13.000	18.725	-25.095	
	Equal variances not assumed			.669	10.915	.517	13.000	19.421	-29.787	
LDL, mg/dl	Equal variances assumed	.612	.440	.195	33	.847	3.324	17.068	-31.401	
	Equal variances not assumed			.172	9.777	.867	3.324	19.381	-39.994	
HDL, mg/dl	Equal variances assumed	1.516	.227	.211	33	.834	1.7991	8.5230	-15.5410	
	Equal variances not assumed			.306	25.942	.762	1.7991	5.8846	-10.2983	
TG, mg/dl	Equal variances assumed	4.192	.049	1.452	33	.156	64.431	44.381	-25.863	
	Equal variances not assumed			2.342	32.437	.025	64.431	27.506	8.432	
Fibrinogen, g/l	Equal variances assumed	.237	.630	-.689	33	.495	-.36593	.53086	-1.44596	
	Equal variances not assumed			-.816	15.552	.427	-.36593	.44856	-1.31907	
CLT, min	Equal variances assumed	.835	.367	1.352	33	.186	16.019753086419755	11.850089983389951	-8.089436260935436	40.12894
	Equal variances not assumed			1.546	14.477	.144	16.019753086419755	10.364073395602674	-6.140457603520552	38.17996
Ks, 10^-9cm^2	Equal variances assumed	.040	.842	.421	33	.677	.428428988862202	1.018805533347057	-1.644346453858174	2.50120

IBM SPSS Web Report - Microclot Cont. Variables Analysis.spv

	Equal variances not assumed			.061	19.842	.952	.00093	.01514	-.03068
Lagtime_(min)	Equal variances assumed	.001	.977	-.945	33	.352	-.77185	.81684	-2.43372
	Equal variances not assumed			-1.020	13.002	.326	-.77185	.75650	-2.40615
ETP_(nM•min)	Equal variances assumed	2.087	.158	2.400	33	.022	498.66347	207.74482	76.00347
	Equal variances not assumed			2.740	14.425	.016	498.66347	182.01924	109.34803
Peak_(nM)	Equal variances assumed	.424	.520	1.806	33	.080	96.31185	53.32347	-12.17557
	Equal variances not assumed			2.009	13.729	.065	96.31185	47.92943	-6.67750
ttPeak_(min)	Equal variances assumed	1.201	.281	-1.587	33	.122	-1.83593	1.15703	-4.18992
	Equal variances not assumed			-1.412	9.891	.189	-1.83593	1.30013	-4.73715
PAI-I, ng/ml	Equal variances assumed	.404	.529	1.237	33	.225	7.9718917	6.4452774	-5.1411238
	Equal variances not assumed			1.459	15.443	.165	7.9718917	5.4639019	-3.6450981
tPA Ag, ng/ml	Equal variances assumed	.449	.508	.042	33	.967	.0931287	2.2276268	-4.4390120
	Equal variances not assumed			.039	10.449	.970	.0931287	2.3895229	-5.2002177
TAFI act, %	Equal variances assumed	.057	.813	.777	33	.443	2.798005	3.601167	-4.528624
	Equal variances not assumed			.858	13.532	.406	2.798005	3.262021	-4.221106
TAFI Ag, %	Equal variances assumed	.031	.862	.397	33	.694	3.2389593	8.1623912	-13.3675506
	Equal variances not assumed			.363	10.227	.724	3.2389593	8.9109645	-16.5563947
a2AP, %	Equal variances assumed	.032	.858	.731	33	.470	4.157	5.688	-7.416
	Equal variances not assumed			.677	10.367	.513	4.157	6.141	-9.460
Plasminogen, %	Equal variances assumed	3.812	.059	1.429	33	.162	8.250	5.774	-3.497
	Equal variances not assumed			1.986	23.212	.059	8.250	4.154	-.339
Lp(a), mg/dl	Equal variances assumed	.085	.772	.341	33	.736	4.1486	12.1814	-20.6347
	Equal variances not assumed			.360	12.531	.725	4.1486	11.5269	-20.8487
proBNP, pg/ml	Equal variances assumed	.056	.814	-.060	33	.952	-110.542	1834.164	-3842.176
	Equal variances not assumed			-.082	22.102	.935	-110.542	1343.595	-2896.244
TnT, pg/ml	Equal variances assumed	8.006	.008	-1.894	33	.067	-38.0792	20.1086	-78.9904
	Equal variances not assumed			-1.324	8.039	.222	-38.0792	28.7506	-104.3219
CRP, mg/l	Equal variances assumed	.882	.355	.031	33	.975	.46380	14.91125	-29.87336
	Equal variances not assumed			.026	9.273	.980	.46380	17.82354	-39.67539
D-dimer, ng/ml	Equal variances assumed	1.887	.179	-.386	33	.702	-904.68278	2343.17014	-5671.89828
	Equal variances not assumed			-.552	25.037	.586	-904.68278	1639.42770	-4280.89704
P-selektyna, ng/ml	Equal variances assumed	.476	.495	-1.085	33	.286	-11.37444	10.48041	-32.69700
	Equal variances not assumed			-1.185	13.271	.257	-11.37444	9.59505	-32.06031
PF4, ng/ml	Equal variances assumed	.004	.950	-1.927	33	.063	-11.71532	6.08024	-24.08567
	Equal variances not assumed			-1.813	10.574	.098	-11.71532	6.46063	-26.00519
Hcy, uM	Equal variances assumed	4.022	.053	.844	33	.405	9.7338	11.5392	-13.7428
	Equal variances not assumed			1.495	30.573	.145	9.7338	6.5125	-3.5560
AT, %	Equal variances assumed	.023	.880	-.511	33	.613	-2.875	5.628	-14.325
	Equal variances not assumed			-.498	11.067	.629	-2.875	5.779	-15.584
PC, %	Equal variances assumed	.016	.900	-.290	33	.773	-3.116	10.728	-24.942
	Equal variances not assumed			-.285	11.189	.781	-3.116	10.928	-27.119
PS, %	Equal variances assumed	.860	.360	1.507	33	.141	13.532	8.980	-4.737
	Equal variances not assumed			1.593	12.534	.136	13.532	8.496	-4.891
FVIII, %	Equal variances assumed	.440	.512	.882	33	.384	17.005	19.272	-22.206
	Equal variances not assumed			.986	13.846	.341	17.005	17.245	-20.020

IBM SPSS Web Report - Microclot Cont. Variables Analysis.spv

B2GPI IgM	Equal variances assumed	.662	.422	.593	33	.557	4.7060	7.9299	-11.4275	
	Equal variances not assumed			1.029	31.989	.311	4.7060	4.5747	-4.6126	
aCL IgG	Equal variances assumed	.017	.896	-.432	33	.668	-.5644	1.3058	-3.2210	
	Equal variances not assumed			-.477	13.527	.641	-.5644	1.1830	-3.1101	
aCL IgM	Equal variances assumed	1.390	.247	.797	33	.431	3.5005	4.3924	-5.4360	
	Equal variances not assumed			1.343	32.913	.188	3.5005	2.6061	-1.8021	
Max OD	Equal variances assumed	4.645	.039	-.804	33	.427	-.065139	.080981	-.229896	
	Equal variances not assumed			-1.169	26.160	.253	-.065139	.055736	-.179671	
Lag Time	Equal variances assumed	6.292	.017	-.803	33	.428	-52.630	65.559	-186.011	
	Equal variances not assumed			-.483	7.397	.643	-52.630	109.026	-307.658	
Time to Max OD	Equal variances assumed	1.978	.169	-.844	33	.405	-844.796	1001.077	-2881.503	
	Equal variances not assumed			-.688	9.051	.509	-844.796	1227.755	-3619.768	
Average Rate of Clotting	Equal variances assumed	.104	.749	.476	33	.637	.000074778	.000157064	-.000244771	
	Equal variances not assumed			.574	16.183	.574	.000074778	.000130334	-.000201263	
Maximum Rate of Clotting	Equal variances assumed	.015	.902	-.030	33	.976	-.000008718	.000291436	-.000601649	
	Equal variances not assumed			-.031	12.165	.976	-.000008718	.000280816	-.000619643	
Fibers / 100 um	Equal variances assumed	.737	.397	-1.117	31	.272	-1.90795	1.70782	-5.39107	
	Equal variances not assumed			-1.255	14.673	.229	-1.90795	1.51982	-5.15367	
2Max OD	Equal variances assumed	1.301	.262	.227	33	.822	.018819	.082767	-.149570	
	Equal variances not assumed			.276	16.415	.786	.018819	.068249	-.125564	
deltaLysis	Equal variances assumed	1.506	.230	-.151	29	.881	-162.800	1079.549	-2370.727	
	Equal variances not assumed			-.249	22.248	.806	-162.800	653.349	-1516.887	
delta OD	Equal variances assumed	.157	.695	-1.057	33	.298	-.083958333333333	.079405934391117	-.245510921560300	.077594[illegible]
	Equal variances not assumed			-1.131	12.794	.279	-.083958333333333	.074224120002054	-.244572419530571	.076655[illegible]
