# Supplementary material for: Plasma from patients with pulmonary embolism show aggregates that reduce after anticoagulation
Source: Commun Med (Lond). 2023 Jan 28;3:12. doi: 10.1038/s43856-023-00242-8 (PMC9883810; doi:10.1038/s43856-023-00242-8)
Supplement: Supplementary file 5 — Description of Additional Supplementary Files [file 43856_2023_242_MOESM5_ESM.pdf]

Description of Additional Supplementary Files

Supplementary Data 1

Laser scanning confocal images of blood plasma aggregates after clotting with 0.1 U/ml thrombin and 10 mM $CaCl_2$ (slides 1-21), and prior to the addition of thrombin and (slides 23-31). Alexa Fluor 488 labeled fibrinogen is shown green and DiOC6(3) staining is shown in red. Scanning electron micrographs (magnifications between 5,000 and 50,000x) of blood plasma aggregates after clotting with 0.1 U/ml thrombin and 10 mM $CaCl_2$ (slides 33-37).

Supplementary Data 2

Comparisons of categorical clinical parameters (e.g. gender, presence of other disease, drug treatments etc.) between patients with and without plasma aggregates by Chi Square tests using SPSS statistical software package.

Supplementary Data 3

Comparisons of continuous clinical parameters (e.g. age, BMI, cell counts, lipids, protein concentrations etc.) between patients with and without plasma aggregates by independent samples t-Tests using SPSS statistical software package.
